# Role of HOXA1-4 in the development of genetic and malignant diseases

**DOI:** 10.1186/s40364-024-00569-x

**Published:** 2024-02-05

**Authors:** Lumin Wang, Haifeng Sun, Li Cao, Jinhai Wang

**Affiliations:** 1 Gastroenterology Department, The Second Affiliated Hospital of Xi’an Jiaotong University, Xi’an, People’s Republic of China; 2 The Third Department of Medical Oncology, Shaanxi Provincial Cancer Hospital Affiliated to Medical College of Xi’an Jiaotong University, Xi’an, Shaanxi People’s Republic of China; 3 Department of Cell Biology and Genetics, School of Basic Medical Sciences, Xi’an Jiaotong University Health Science Center, Xi’an, Shaanxi People’s Republic of China

**Keywords:** Transcription factors, HOXA1-4, Biomarkers, Malignant diseases

## Abstract

The HOXA genes, belonging to the HOX family, encompass 11 members (HOXA1-11) and exert critical functions in early embryonic development, as well as various adult processes. Furthermore, dysregulation of HOXA genes is implicated in genetic diseases, heart disease, and various cancers. In this comprehensive overview, we primarily focused on the HOXA1-4 genes and their associated functions and diseases. Emphasis was placed on elucidating the impact of abnormal expression of these genes and highlighting their significance in maintaining optimal health and their involvement in the development of genetic and malignant diseases. Furthermore, we delved into their regulatory mechanisms, functional roles, and underlying biology and explored the therapeutic potential of targeting HOXA1-4 genes for the treatment of malignancies. Additionally, we explored the utility of HOXA1-4 genes as biomarkers for monitoring cancer recurrence and metastasis.

## Introduction

**Fig. 1:**
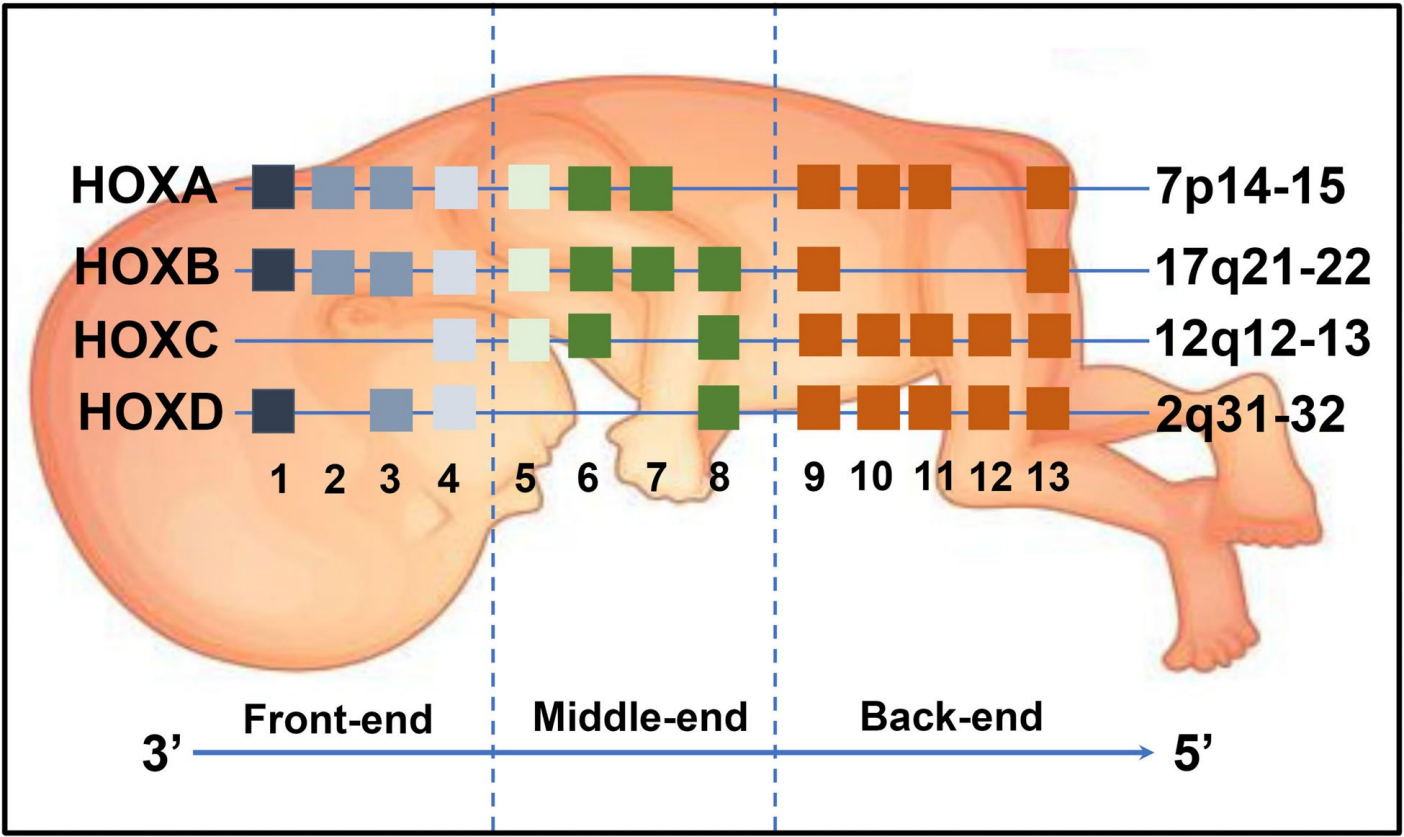
The human HOX gene clusters. 39 HOX are organized into four chromosomal clusters: HOXA, HOXB, HOXC, and HOXD

Homeobox (HOX) genes are a group of evolutionarily conserved transcription factors, which can be categorized into four family clusters: HOXA, HOXB, HOXC, and HOXD (Fig. 1). These genes play crucial roles in cellular development and the establishment of body patterns during embryogenesis. For instance, mutations in HOXA13 have been associated with the absence of thumb formation [1]. Similarly, heterozygous alterations in HOXD13 are linked to Synpolydactyly 1, an inherited limb deformity [2]. Furthermore, HOX genes have been implicated in the progression of neurological disorders. Notably, the HOXD subset is critically involved in the development of monosynaptic sensory-motor connections. In mice, the loss of Hoxd9, Hoxd10, and Hoxd11 results in locomotion defects [3]. Moreover, the regulation of HOXB3 by LncRNA147410.3 influences crucial physiological processes, such as proliferation, differentiation, and apoptosis, in mouse microglia [4]. Dysregulated methylation levels affecting HOXB4, HOXC4, and HOXD1 have been associated with neural tube defects (NTDs) [5]. Additionally, abnormal expression patterns of HOX genes have been observed in various diseases, including hypertensive disorder complicating pregnancy (HDCP) [6] and allergic asthma [7]. For example, miR-1233 directly targets HOXB3, suppressing trophoblast cell invasion, and contributing to HDCP. Furthermore, HOX5 regulates the function of Th2 cells in chronic allergic inflammation by modulating Gata3.

Previous research has revealed the regulatory role of HOX genes in human cancer development, affecting processes such as cell proliferation, differentiation, apoptosis, metastasis, angiogenesis, and epithelial-mesenchymal transition (EMT). While some HOX genes are reported to function as tumor suppressors, recent studies suggest that HOX genes primarily act as oncogenes in cancer progression. For example, HOXD1 is downregulated in kidney renal clear cell carcinoma (KIRC), and its high expression is associated with the inhibition of cancer cell proliferation, cell cycle progression, and TGF-β signaling [8]. Similarly, both loss-of-function and gain-of-function experiments have demonstrated that HOXD13 inhibits cell proliferation and migration in prostate cancer (PCa) by blocking BMP4/SMAD1 signaling [9]. Conversely, HOXB3/6/7/8/9 are highly expressed in lung adenocarcinoma (LUAD) and correlate with poor overall survival (OS) [10]. Additionally, HOXC6/8/9/10/11/13 are overexpressed in gastric cancer (GC) and are associated with an unfavorable prognosis [11]. Our previous research discovered that HOXD3 acted as an oncogene, promoting the proliferation, metastasis, invasion, and angiogenesis of liver cancer through the ITGA and EGFR pathways [12, 13].

HOXA genes, a specific subset within the HOX family, have received significant attention in the fields of organ development, disease pathogenesis, and various cancer types. These genes have been identified as crucial players in cancer progression, acting both as oncogenes and inhibitors. For instance, HOXA5 has been implicated in the regulation of vascular smooth muscle cell phenotype, thereby exerting a protective effect against carotid atherosclerosis [14]. In GC, co-expression of HOXA6 with PBX2 has been observed, resulting in enhanced cell proliferation, metastasis, and invasion [15]. However, conflicting findings suggest that HOXA6 actually acts as an inhibitor, suppressing cell proliferation through the PI3K/AKT signaling pathway in clear cell renal cell carcinoma [16].

Gaining a comprehensive understanding of the involvement of HOXA genes in the development of HOXA-related diseases is crucial for identifying novel treatment targets and innovative therapeutic strategies. Therefore, the objective of this study is to provide an extensive review encompassing the diverse functions of HOXA1-4 genes in the pathogenesis of both inherited and acquired diseases. This will be achieved through an evaluation of their regulatory mechanisms, biological functions, and associated pathways. Additionally, we aim to consolidate preliminary data that support the potential use of these genes as targets for gene therapy, offering new perspectives for the treatment of HOXA-related diseases.

### Structural domains of HOXA1-4

Mammals possess a total of 39 HOX genes, which are organized into four chromosomal clusters: HOXA (7p15-p14), HOXB (17q21-q22), HOXC (12q12-q13), and HOXD (2q31-q32). These genes encode transcription factors that contain homeodomains and are vital for the development of vertebral architecture along the anteroposterior axis. Notably, the expression of HOX genes follows a specific pattern, with the front-end genes at the 3' end being expressed first. Consequently, they can activate the expression of the posterior HOX genes located at the 5' end. This sequential expression leads to temporal and spatial collinearity among the HOX genes within each cluster. Thus, the regulatory interactions among HOX genes during embryonic development ultimately determine the morphology of body segments.

The HOXA genes are arranged in a sequential manner based on their paralog groups, which span from 1 to 13. The HOXA gene cluster comprises HOXA1, HOXA2, HOXA3, HOXA4, HOXA5, HOXA6, HOXA7, HOXA9, HOXA10, HOXA11, and HOXA13. Remarkably, the genes HOXA1-4 significantly influence the development of the head in mammals (Fig. 1).

### Different roles of HOXAs in common diseases

#### HOXA1

**Fig.2:**
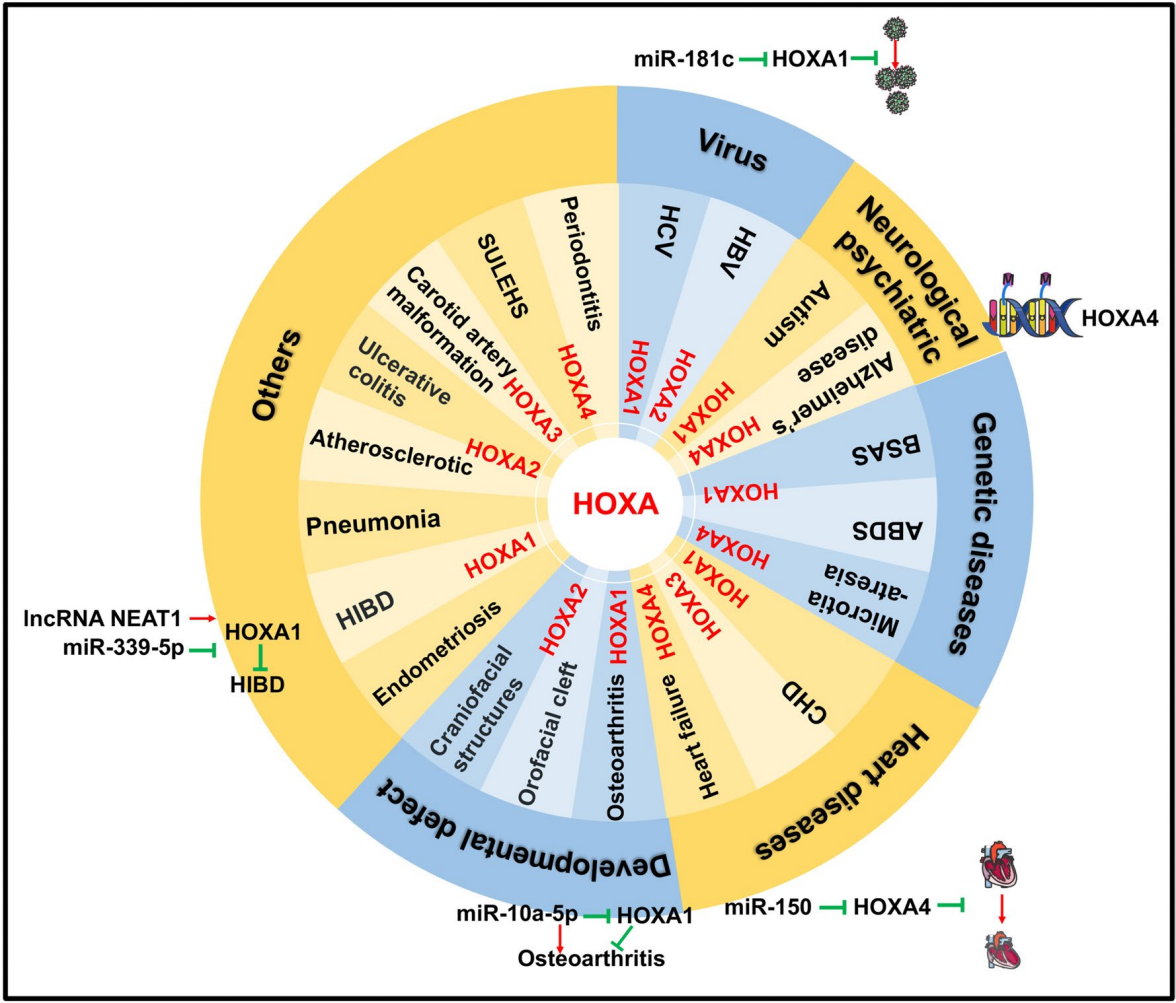
HOXA1-4 genes have participated in the regulation of gene expression related to the heart, blood vessels, viruses, genetic diseases, neurology, psychiatry, and developmental defects

The crucial role played by HOXA genes in cell physiology is evident in the numerous diseases that arise as a result of gene mutations or altered expression (Fig. 2). For instance, mutations in HOXA1 have been linked to a group of genetic disorders referred to as "HOXA1-related syndromes" [17]. The phenotypic manifestations of these syndromes can vary across different populations. One of the examples is Bosley-Salih-Alorainy Syndrome [18], which has been reported predominantly among Middle Eastern patients. Characteristic symptoms of this syndrome include facial weakness, hearing impairment, sensorineural hearing loss, cardiac malformations, and developmental delays. Another illustration is Athabascan Brain Dysgenesis Syndrome (ABDS), observed primarily in the Native American population. Individuals affected by ABDS exhibit features such as mental retardation, central hypoventilation, facial weakness, and conotruncal heart defects [19, 20]. Additionally, a truncated mutation of HOXA1 has been identified in pigs with congenital malformation of the outer ears, discovered through RNA sequencing analysis of pig embryos. Given that the incidence rate of diseases in pigs is similar to that in humans, this finding can act as a reference point for comprehending the molecular mechanisms underlying genetic diseases in humans [21].

In recent years, several studies have provided evidence of the involvement of HOXA1 expression in heart disease, particularly in congenital heart defects (CHD) [22]. For instance, investigations have revealed that variations in the poly-histidine repeat motif of HOXA1 can induce the development of bicuspid aortic valve (BAV) in mice and zebrafish [23]. BAV represents the most common form of CHD and is characterized clinically by various abnormalities in the aortic valve, including aortic stenosis and regurgitation, endocarditis, and ascending aortic aneurysm/dissection, affecting approximately one-third of patients. Furthermore, HOXA1 is expressed in precursors of cardiac neural crest cells (NCCs) and contributes to heart development through the regulation of NCCs [24].

HOXA1 not only plays a crucial role in heart disease regulation but is also involved in joint-related diseases, specifically through its influence on apoptosis. Increased levels of miR-10a-5p promote chondrocyte apoptosis by suppressing HOXA1 expression in osteoarthritis [25]. This finding has been further supported by miR-18a-3p, which targets the 3' UTR of HOXA1, thus inhibiting its expression and inducing chondrocyte apoptosis [26].

In studies pertaining to autism, researchers have discovered a correlation between the HOXA1 A218G polymorphism and variations in head circumference among autistic patients [27]. Additionally, Ingram et al. have determined that the presence of a (His)73(Arg) polymorphism (A:G) in the HOXA1 gene significantly contributes to the susceptibility of autism [28]. However, these findings have been called into question by the Dr. Devlin group, who conducted an analysis of genotype frequencies and allele transmissions using data from the Collaborative Programs of Excellence in Autism network. Their results suggest that the (His)73(Arg) polymorphism in the HOXA1 gene does not have a significant association with autism [29].

HOXA has demonstrated functional involvement in various diseases, such as endometriosis [30], hypoxic-ischemic brain damage (HIBD) [31], Hepatitis C virus (HCV), and sepsis-induced pneumonia [32]. In endometriosis, all HOXA and HOXB paralogs, except for HOXA1, exhibited significant down-regulation in ectopic tissues compared to control tissues. In the case of HIBD, the long non-coding RNA NEAT1 competes with miR-339-5p to effectively increase HOXA1 expression, promoting neuronal cell viability and suppressing apoptosis during hypoxia–ischemia. Similarly, in HCV research, the expression of HOXA transcript antisense RNA myeloid-specific 1 (HOTAIRM1) is upregulated, suggesting its potential as an immunomodulatory target that regulates the expressions of HOXA1 and miR-124 and promotes the expansion of myeloid-derived suppressor cells [33]. Correspondingly, miR-181c targets HOXA1 and, through the regulation of STAT3 and STAT5, hampers HCV replication [34]. Lastly, in sepsis-induced pneumonia, the decreased expression of urothelial carcinoma-associated 1 (UCA1) leads to the downregulation of EZH2, consequently increasing HOXA1 expression. This upregulation of HOXA1 contributes to the mitigation of sepsis-induced pneumonia progression.

### HOXA2

Increasing evidence supports the involvement of epigenetic regulation in various diseases related to HOXA2. For example, a DNA methylation analysis revealed that HOXA2 exhibits hypomethylation in carotid atherosclerotic plaques, in contrast to intact tissues from internal mammary arteries and saphenous veins [35]. The impact of HOXA2 on fibrosis progression in chronic hepatitis B infection was confirmed using Illumina Infinium Bead Arrays. Findings from these studies indicate a significant association between hypermethylation of HOXA2 and severe fibrosis [36]. In the investigation of orofacial cleft, distinct methylation patterns were observed for different subtypes of orofacial cleft within the HOXA2 gene [37]. Additionally, the long non-coding RNA HOTAIRM1 was found to suppress DNMT1 enrichment on the HOXA2 promoter, resulting in hypomethylation and subsequent induction of HOXA2 expression, ultimately leading to osteogenesis of human periodontal ligament stem cells [38].

Furthermore, the expression of HOXA2 has been linked to ulcerative colitis (UC). HOXA2 was found to be significantly downregulated in UC patients compared to both remission patients and healthy controls [39]. Moreover, HOXA2 has also been associated with congenital malformations of the external ear. Utilizing Phenolyzer, a phenotypic-based gene analyzer, computational analysis has confirmed this relationship [40]. Supporting this finding, Si et al. discovered that HOXA2 expression is connected to the evolutionarily conserved enhancer region of the homeobox 1 transcription factor gene (HMX1), which plays a role in craniofacial structure development [41]. Additionally, HOXA2 is involved in the progression of chondrogenesis and has been implicated in human idiopathic proportionate short stature due to its dysregulation [42].

### HOXA3

In wound healing, research has revealed that HOXA3 promotes neovascularization to expedite the wound healing. This effect is achieved by mobilizing, recruiting, and enhancing the differentiation of multipotent bone marrow-derived cells, while concurrently inhibiting the expression of proinflammatory nuclear factor kappa B pathway-related proteins [43]. Similarly to HOXA1, HOXA3 also plays a critical role in CHD development [22]. HOXA3 exhibits distinct expression patterns within sub-domains of second heart field cells, which contribute to embryonic heart development during cardiac looping [44]. The absence of the third arch artery, resulting in carotid artery system malformation, has been observed in homozygous HOXA3 mutants [45, 46]. Moreover, HOXA3 has been associated with epigenetic modifications. Differential hypermethylation in HOXA3 has been linked to neurodegeneration in late-onset Alzheimer's disease (AD) [47]. Interestingly, HOXA3 methylation has also been correlated with physical activity, as a study demonstrated that reduced physical activity in young adults is associated with an upregulation of HOXA3 methylation [48].

### HOXA4

The expression of HOXA4 has been implicated in heart failure (HF). In patients with HF, the long noncoding RNA Myocardial Infarction-Associated Transcript (MIAT) exhibits upregulation, thereby enhancing the expression of HOXA4 through the suppression of miR-150. This contributes to the progression of HF [49]. Additionally, HOXA4 plays a crucial role in maintaining spatial identity within the adult aorta, and its upregulation can decrease susceptibility to human abdominal aortic aneurysms [50]. Likewise, studies conducted by Kimura et al. utilizing a HOXA4-deficient mouse model have shown that HOXA4 interacts with Transcriptional Enhancer Activator Domain (TEADs), leading to the attenuation of Yes-Associated Protein (YAP)/TEAD-mediated transcription by competing for TEAD binding. This interaction affects the phenotypic switching of vascular smooth muscle cells [51]. Furthermore, HOXA4 has the ability to induce the differentiation of human umbilical cord mesenchymal stem cells into epidermal-like cells, thereby promoting skin repair [52].

In the investigation of genetic-related diseases, abnormal expression of HOXA4 has been observed, with mutations in HOXA4 being associated with microtia-atresia, a rare congenital condition [53]. Additionally, HOXA4 is involved in epigenetic modifications. The expression of HOXA4 is inhibited by TASP1, a heterodimeric endopeptidase that activates histone methyltransferases of the KMT2 family. Loss-of-function variants of TASP1 can lead to Suleiman-El-Hattab syndrome (SULEHS), a disorder affecting histone modification [54]. Moreover, alterations in DNA methylation at cytosine-phosphate-guanine (CpG) sites within HOXA4 have been linked to both AD [55] and periodontitis [56]. The increased mRNA level of HOXA4 has also been correlated with hypospadias [57].

The information presented highlights the pivotal involvement of CpG methylation and gene mutations in the onset and progression of diseases influenced by HOXA genes. Additionally, the functions attributed to HOXA1-4 primarily revolve around the regulation of gene expression associated with the heart, blood vessels, viruses, inflammation, neurology, psychiatry, and developmental anomalies. Consequently, a comprehensive investigation of HOXA1-4 can offer valuable insights into the molecular biology of these diseases, thus providing potential research directions and targets for their development.

### Role of HOA1-4 genes in the progression of cancer

**Fig. 3:**
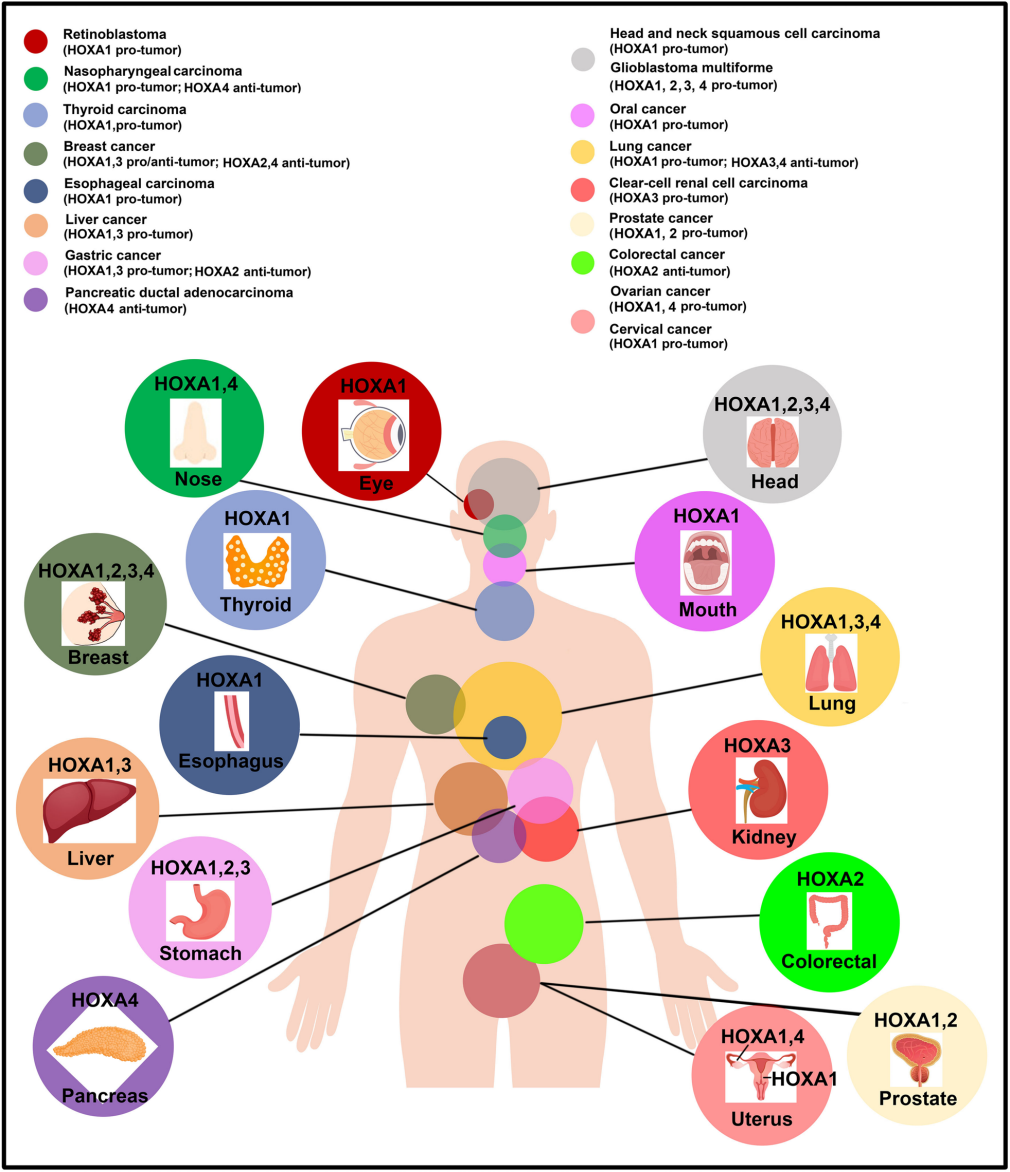
HOXA1-4 genes dysregulation in human cancers. The color code highlights alterations found for more than one HOXA gene in different tumor sites

The pivotal involvement of HOXA1-4 in the development and progression of diverse cancer types has been firmly established, as illustrated in Fig. 3. Numerous studies have consistently shown that the activation of HOXA1-4 genes can drive cancer progression. However, it is important to acknowledge that in certain tumor types, the impact of HOXA1-4 may exhibit an opposing effect, with their expression acting as a tumor suppressor.

### Hepatocellular carcinoma (HCC)

Previous evidence has demonstrated that HOXA1 can serve as a methylation biomarker to improve sensitivity in the early-stage detection of HCC [58–61]. Meanwhile, numerous studies have indicated the regulation of HOXA1 and HOXA3 proteins by non-coding RNAs in HCC (Table 1). For instance, miR-218 and miR-99a directly bind to the putative 3'-UTR binding sites of HOXA1, inhibiting its expression levels and consequently suppressing the proliferation, invasion, and metastasis of HCC [62, 63]. Additionally, the H3K9 demethylase, KDM3A, is targeted by miRNA-202-3p and enhances expression of the HOXA1 by erasing the H3K9me2 to increase the growth of human HCC cell [64]. Moreover, the lncRNA HOXA-AS2 drives the malignant behavior of hepatoblastoma by upregulating HOXA3 expression [65]. Circular RNAs, as non-coding RNAs, also play a role in liver cancer progression. Knockdown of circWHSC1 has been shown to suppress the proliferation and metastasis of liver cancer cell lines by regulating HOXA1. Molecular studies have revealed that circWHSC1 functions as a sponge for miR-142-3p, which targets HOXA1 [66].

**Table 1:** HOXA1-4 genes were regulated by ncRNA in cancers

ncRNA	Tumor	HOXAs	Direct/Indirect	HOXA up/low expression	Function	References
**miRNAs**						
miR-10a-5p	OC	HOXA1	Direct	Up	Viability, colony formation, migration ability, invasiveness	[67]
miR-10b	CC	HOXA1	Direct	Up	Proliferation, invasion	[68]
miR-10b	ccRCC	HOXA3	Direct	Up	Invasion, migration	[69]
miR-100	NSCLC	HOXA1	Direct	Up	Invasion, migration	[70]
miR-100	NPC	HOXA1	Direct	Up	Proliferation	[71]
miR-202-3p	HCC	HOXA1	Indirect	Up	Viability, migration, invasion	[64]
miR-218	HCC	HOXA1	Direct	Up	Proliferation	[62]
miR-218	MDS	HOXA1	Direct	Up	Proliferation, colony formation, and apoptosis	[72]
miR‑338‑3p	T‑LBL	HOXA3	Direct	Up	Malignancy	[73]
miR‑577	NSCLC	HOXA1	Direct	Up	Proliferation, invasion	[74]
miR-99a	HCC	HOXA1	Direct	Up	Invasion, migration	[63]
miR-99a	OC	HOXA1	Direct	Up	Proliferation,invasion	[75]
**lncRNAs**						
DLX6‐AS1	TC	HOXA1	Direct as a sponge of miR‐193b‐3p	Up	Autophagy, apoptosis	[76]
HOTAIR	SCLC	HOXA1	Indirect By H3K27me3	N/A DNA methylation	Multidrug resistance	[77]
HOTAIRM1	GBM	HOXA1	Demethylation of histone H3K9 and H3K27	Up	Proliferation, migration, invasion	[78]
HOTAIRM1	EC	HOXA1	N/A	Up	Proliferation, migration, invasion	[79]
HOXA-AS2	HB	HOXA3	Form RNA‐RNA dimers and increase the stability of HOXA3	Up	Proliferation, migration, invasion	[65]
HOXA-AS3	NSCLC	HOXA3	Direct	Down	EMT, cisplatin resistance	[80]
LINC00152	CC	HOXA1	Direct as a sponge of miR-216b-5p	Up	Proliferation	[81]
LINC00689	GC	HOXA3	Direct as a sponge of miR-338-3p	Up	Cell growth, EMT	[82]
LINC00958	LUAD	HOXA1	N/A	Up	Proliferation, migration	[83]
SNHG1	BC	HOXA1	Direct as a sponge of miR-193a-5p	Up	Proliferation, migration	[84]
ZFPM2-AS1	RB	HOXA1	Direct as a sponge of miR‐515	Up	Growth, metastasis	[85]
**circRNAs**						
circ_0058063	Esophageal cancer	HOXA1	Direct as a sponge of miR-377-3p	Up	Proliferation, migration, invasion,	[86]
circ_0074032	PCa	HOXA1	Direct as a sponge of miR-198	Up	Proliferation, migration, invasion	[87]
circ_0008945	BC	HOXA3	Direct as a sponge of miR-338-3p	Up	Proliferation, migration, invasion	[88]
circEIF4G2	CC	HOXA1	Direct as a sponge of miR‑218	Up	proliferation, migration, invasion	[89]
circRASSF2	BC	HOXA1	Direct as a sponge of miR-1205	Up	Proliferation, migration	[90]
circWHSC1	HCC	HOXA1	Direct as a sponge of miR-142-3p	Up	Proliferation, migration	[66]

*BC* Breast cancer, *CC* Cervical cancer, *ccRCC* Clear cell renal cell carcinoma, *EC* Endometrial cancer, *GBM* Glioblastoma multiforme, *GC* Gastric cancer, *HB* Hepatoblastoma, *HCC* Hepatocellular carcinoma, *lncRNA* Long non-coding RNA, *LUAD* Lung adenocarcinoma, *MDS* myelodysplastic neoplasm, *miRNA* microRNAs, *ncRNA* non-coding RNAs, *NPC* Nasopharyngeal carcinoma, *NSCLC* Non‑small cell lung cancer, *OC* Ovarian cancer, *PCa* Prostate cancer, *RB* Retinoblastoma, *SCLC* Small cell lung cancer, *TC* Thyroid carcinoma, *T‑LBL* T‑cell lymphoblastic lymphoma

### Lung cancer

Multi-database joint analysis has revealed the upregulation of HOXA1 expression in non-small cell lung cancer (NSCLC). These databases, namely Oncomine, Gene Expression Profiling Interactive Analysis, the Multi Experiment Matrix, and The Cancer Genome Atlas (TCGA), have provided supporting evidence [91]. In contrast, the TCGA database has shown that HOXA3 expression is decreased in NSCLC tissues, and this finding has been verified through RT-PCR assay in clinical samples [92]. Additionally, studies conducted by Tang and Gao et al. have demonstrated that HOXA4, similar to HOXA3, functions as a tumor suppressor in the progression of lung cancer [93, 94].

The identification of DNA hypermethylation at promoter CpG islands has emerged as a critical factor in lung cancer progression. Notably, HOXA1 has shown highly significant hypermethylation in lung cancer, which holds promise as a potential marker for early detection of LUAD [95]. The hypermethylation of HOXA1 may be regulated by H3K27me3 through HOTAIR, thereby contributing to the development of chemoresistance in small-cell lung cancer (SCLC) [77]. Aberrant methylation of HOXA1 has also been observed in preinvasive lesions, which are implicated in the early stages of pulmonary adenocarcinoma (ADC) development [96]. Similarly, hypermethylation of CpG sites in the HOXA3/HOXA4 region has been detected in lung cancer [97].

A growing body of evidence indicates that the dysregulation of non-coding RNAs (ncRNAs) in conjunction with HOXA plays a regulatory role in lung cancer progression. For instance, miR-577 directly targets HOXA1, and the functional effect of miR-577 on lung cancer cells is contingent upon HOXA1 [74]. Knockdown of oncogenic lncRNA LINC00958 leads to the inhibition of an oncogenic phenotype, which can be restored by HOXA1 overexpression in LUAD [83]. Furthermore, HOXA3 is regulated by LncRNA HOXA-AS3, an oncogene that enhances cisplatin resistance and promotes EMT [80]. The role of HOXA extends beyond tumor proliferation, migration, and invasion, as it also contributes to immune evasion in lung cancer. Studies have demonstrated notable associations between HOXA1 and immune cell infiltration as well as immune checkpoints. Knockdown of HOXA1 in LUAD cells enhances the CD8^+^ T cell response [98].

### Breast cancer (BC)

In a study using RT-PCR assay to analyze HOX gene expression in human invasive ductal BC tissues and normal tissues, significant differences in expression were observed for 11 HOX genes (HOXA1, A2, A3, A5, A9, C11, D3, D4, D8, D9, and D10) between cancerous and normal tissues. Specifically, HOXA1, A2, and A3 showed lower expression in cancerous tissues compared to normal tissues [99]. The functional relevance of HOXA1 in BC was also confirmed by Magali Belpaire et al. [100]. Additionally, a downregulation of HOXA4 was observed in BC patients compared to non-cancerous tissues, which may be attributed to increased DNA methylation levels of HOXA4 [101].

Contrasting these findings, some research suggests that HOXA1 acts as an oncogene. Liu et al. demonstrated that both mRNA and protein levels of HOXA1 were increased in BC. High expression of HOXA1 was associated with poor prognosis and advanced clinicopathological features in BC patients. Inhibition of HOXA1 significantly suppressed cell progression by enhancing cell apoptosis and inducing cell cycle arrest in BC cells [102].

Moreover, the regulation of HOXA1 and HOXA3 by non-coding RNAs plays a role in modulating BC progression. Overexpression of circRASSF2 increased HOXA1 protein expression, leading to enhanced abilities of proliferation, clone formation, invasion, and metastasis in BC cells [90]. LncRNA SNHG acts as a sponge for miR-193a-5p, activating the expression of HOXA1 [84]. In the case of HOXA3, overexpression of miR-338-3p downregulates HOXA3 expression, whereas knockdown of miR-338-3p increases HOXA3 expression [88].

### Prostate cancer

PCa is a significant disease primarily affecting men. Studies have demonstrated that HOXA1 overexpression promotes the growth, invasion, and metastasis of PCa. Conversely, suppression of HOXA1 results in high expression of E-cadherin and low expression of Snail and MMP-3 in PCa cells, indicating a potential role in inhibiting cancer progression [103]. Additionally, non-coding RNAs (ncRNAs) play crucial roles in PCa tumorigenesis. An oncogenic role of HOXA1 has been observed in PCa, as its expression can be enhanced by circ_0074032, which functions as a decoy for miR-198 [87]. Moreover, HOXA2 [104] and HOXA4 [105] have also been implicated in PCa progression. Specifically, HOXA2 has been found to display markedly increased expression in metastatic PCa tumors compared to normal tissue, and it is associated with shorter OS in patients.

### Gastric cancer

GC ranks as the third most common cancer globally. Research has shown that HOXA1 exhibits high expression in GC cells, and its inhibition has been observed to reduce the expression of the cell cycle-related protein cyclin D1 [106]. Moreover, HOXA1 is among the 27 DNA methylation markers that display a higher frequency or level of methylation in GC tissues compared to non-neoplastic mucosae tissues [107]. However, a study investigating HOXA2 revealed lower expression of the HOXA2 gene in GC tumor samples than in normal samples [108]. Furthermore, HOXA3 has been identified as a target gene of miR-338-3p, and the upregulation of LINC00689 suppresses miR-338-3p, resulting in increased expression of HOXA3 in GC cells [82].

### Ovarian cancer (OC)

Analysis of data from the TCGA database has revealed a correlation between the overexpression of HOXA1 and A4 and the advanced Federation of Gynecology and Obstetrics (FIGO) stage in epithelial ovarian cancer (EOC) [109]. Furthermore, miR-10a-5p has been found to inhibit the aggressive phenotypes of OC cells by suppressing the expression of HOXA1 [67]. Notably, the overexpression of HOXA4 is significantly associated with shorter progression-free survival in high-grade serous ovarian carcinoma patients [110]. Additionally, HOXA4 expression is elevated in invasive OC compared to noninvasive OC [111, 112].

### Head and neck squamous cell carcinoma (HNSCC)

Based on bioinformatics analysis utilizing gene co-expression network analysis, evidence suggests a negative correlation between the expression levels of HOXA1 and the survival prognosis of the tumor, implying a significant role in HNSCC development [113]. Moreover, both Gene Set Variation Analysis (GSVA) and Gene Set Enrichment Analysis (GSEA) have successfully linked HOXA1 expression to tumor-associated signaling pathways and cell adhesion junction pathways [114]. These findings receive further support from He et al.'s research [115].

### Oral cancer

Five genes, namely ASCL4, CELSR3, HIST1H3J, HOXA1, and ZFP42, have been identified as being upregulated in oral squamous cell carcinoma (OSCC). These genes are involved in various biological processes, including DNA replication, mismatch repair, and the NOTCH-related signaling pathway [116]. The overexpression of HOXA1 in OSCC has been found to be associated with a poor prognosis [117]. Furthermore, HOXA1, in conjunction with Ki67 as a proliferation marker, has been linked to the development of OSCC [118]. Additionally, the study reports an increase in HOXA2 expression during oral dysplasia but its subsequent repression in oral cancer progression [119].

### Pancreatic ductal adenocarcinoma (PDAC)

In PDAC, a study employed a panel consisting of 13 methylated DNA markers (MDMs), namely AK055957, CD1D, CLEC11A, FER1L4, GH05J042948, GRIN2D, HOXA1, LRRC4, NTRK3, PRKCB, RYR2, SHISA9, and ZNF781, in conjunction with carbohydrate antigen 19–9 (CA19-9), to effectively detect PDAC [120]. Furthermore, upregulation of HOXA4 has been observed in PDAC patients with a more favorable prognosis [121].

### Cervical cancer (CC)

HOXA1 is acknowledged as a tumor activator due to its well-established role in promoting proliferation, migration, invasion, and aerobic glycolysis. Notably, its enrichment in CC has been extensively documented [122–124]. Furthermore, miR-10b has been identified as a regulator of HOXA1, targeting its 3′-UTR region and effectively suppressing CC cell growth and invasion [68]. Additionally, circEIF4G2 [89] and LINC00152 [81] act as sponges for miR‑218 and miR-216b-5p, respectively, thereby contributing to the functional regulation of CC. HOXA1 is targeted by miR‑218 and miR-216b-5p and directly modulates the expression of ENO1 and PGK1, thus promoting glycolysis and inducing cancer progression [125]. Correspondingly, HOXA1 exhibits increased expression in early-stage CC compared to adjacent noncancerous tissue and is associated with an unfavorable prognosis [126].

### Esophageal squamous cell carcinoma (ESCC)

The expression of HOXA1 has been found to be elevated in ESCC cells in comparison to normal cells. Interestingly, the impact of HOXA1 on esophageal carcinoma can be mitigated through the use of antisense oligodeoxynucleotides, which effectively suppress the invasion and metastasis of esophageal carcinoma cells by inhibiting the activation of the PI3K/AKT signaling pathway [127]. Moreover, circ_0058063 exerts its influence on HOXA1 expression by targeting miR-377-3p, ultimately enhancing the proliferation of esophageal cancer [86].

### Glioblastoma (GBM)

HOXA1 plays a pivotal role as a tumor activator in promoting the proliferation, metastasis, and invasion of GBM [128]. This activation mechanism is mediated by the lncRNA-HOTAIRM1 through epigenetic modifications [78]. These findings were confirmed by Xia et al. in their study conducted on GBM stem cells [129]. In the investigation of HOXA3, it was observed that HOXA3 is upregulated in GBM patients and is associated with an unfavorable prognosis. Notably, HOXA3 transcriptionally activates aerobic glycolysis, thereby significantly accelerating tumor progression [130, 131]. Furthermore, HOXA4 has been identified as being upregulated in GBM and glioma cells, with its expression levels strongly correlating with a poor prognosis [132].

### Nasopharyngeal carcinoma (NPC)

MiR-100 functions by downregulating the expression of HOXA1, thereby suppressing the growth of NPC cells [71]. By utilizing GEO and gene methylation profiling data, the investigation has identified the expression levels of HOXA4 in NPC. The findings reveal that HOXA4 is characterized by hypermethylation, leading to its reduced expression, and it plays a crucial role in NPC progression [133].

### Other cancers

Feng et al. discovered the involvement of the DLX6-AS1/miR-193b-3p/HOXA1 axis in thyroid carcinoma (TC). Depletion of DLX6-AS1 suppresses TC cell growth and induces autophagy by upregulating miR-193b-3p and downregulating HOXA1 expression [76]. Notably, the methylation level of HOXA1 is significantly higher in Biliary intraepithelial neoplasia, a precancerous lesion of extrahepatic cholangiocarcinoma (EHC) [134]. Moreover, miR-218 overexpression effectively inhibits the development of myelodysplastic neoplasm (MDS) by regulating cell proliferation, colony formation, and apoptosis both in vitro and in vivo. Mechanistically, miR-218 appears to target HOXA1, which plays a key role in the pathogenesis of MDS [72]. Likewise, overexpression of HOXA1 has been demonstrated to promote tumor facilitative effects, including cell proliferation, metastasis, and invasion, in endometrial cancer (EC) [79]. Additionally, aberrant methylation of HOXA2 has identified it as a potential biomarker for colorectal cancer (CRC) [135, 136]. Several studies have reported that miR-338-3p inhibits the development of T-cell lymphoblastic lymphoma by directly targeting the oncogenic factor HOXA3 through binding to the 3'‑UTR region [73]. Additionally, miR-10b has been found to target HOXA3, leading to the inhibition of proliferation and invasion in ccRCC cells [69].

In conclusion, HOXA1 plays a significant role in the regulation of the development and progression of major cancers that have a substantial impact on human survival, including lung cancer, BC,CRC, GC, and liver cancer. Notably, HOXA1 expression is consistently upregulated in lung cancer, GC, and liver cancer. However, its precise role in BC remains unclear, as some studies suggest its function as an oncogene while others indicate otherwise. HOXA2 has received comparatively less research attention, with its expression being downregulated in BC and GC, and hypermethylation observed in CRC. Furthermore, HOXA3 is upregulated in liver cancer and GC, while it is downregulated in lung cancer. Similar to HOXA1, the role of HOXA3 in BC remains uncertain. Conversely, HOXA4 serves as a tumor suppressor gene in lung cancer and BC.

Compared to HOXA-4, HOXA1 has received substantial attention in the investigation of various other tumors. It serves as an oncogene in CC, EC, GBM, HNSCC, NPC, OC, PCa, and TC, mediating tumor proliferation, migration, invasion, and drug resistance. In contrast, HOXA2 exhibits high expression in PCa. HOXA3 displays overexpression in ccRCC, GBM, and T‑LBL. Moreover, HOXA4 acts as an oncogene in GBM regulation, while inhibiting the biological functions of NPC.

### Role of HOXA-AS2/3 in disease regulation

Long non-coding RNA HOXA cluster antisense RNA 2 and 3 (HOXA-AS2/3) is situated within the HOXA gene cluster. Multiple investigations have consistently shown the participation of HOXA-AS2/3 in the advancement of diverse ailments.

### HOXA-AS2 in diseases

In the study of diabetic nephropathy, the HOXA-AS2/miRNA-302b-3p/TIMP3 axis has been determined to protect against inflammatory response and inhibit proliferation in podocytes, thereby mitigating the progression of diabetic nephropathy [137]. Furthermore, HOXA-AS2 upregulates tipe2 expression by directly targeting miR-17-5p, thereby safeguarding lung tissue from chronic intermittent hypoxia injury [138]. HOXA-AS2 targets miRNA-877-3p, which is upregulated following injury in human aortic vascular smooth muscle cells (HA-VSMCs), leading to increased proliferation and metastasis while suppressing VSMC apoptosis [139]. Through high-throughput mRNA sequencing, Zhu et al. revealed that HOXA-AS2 can inhibit endothelial inflammation by suppressing the NF-κB signaling pathway [140]. Inhibition of HOXA-AS2 can alleviate the progression of epilepsy by regulating the miR-372-3p/STAT3 axis [141]. Additionally, the highly expressed HOXA-AS2 modulates microglial polarization by interacting with the PRC2 complex and epigenetically suppressing PGC-1α, thereby increasing neuroinflammation [142]. Furthermore, the decreased expression of HOXA-AS2 negatively regulates systemic lupus erythematosus through inactivating the ERK signaling pathway [143] (Table 2).

**Table 2:** Role of HOXA- AS2/3 in diseases

Diseases	Expression	Regulate genes	pathways	Functions	References
**HOXA-AS2**					
Chronic intermittent hypoxia (CIH)-induced lung inflammation	Down	miR-17-5p	miR-17-5p/tipe2	Inflammatory regulator	[138]
Diabetic nephropathy	Down	miRNA-302b-3p	miRNA-302b-3p/TIMP3	Inflammation, apoptosis	[137]
Epilepsy	Up	miR-372-3p	miR-372-3p/STAT3	Cellular damages	[141]
Parkinson's disease	Up	PRC2, PGC-1α		Neuroinflammation	[142]
Systemic lupus erythematosus	Up		ERK pathway		[143]
Vascular disorders			NF-κB signaling	Endothelium inflammation	[140]
VSMCs injury	Up	miRNA-877-3p		Proliferation, apoptosis, and migration	[139]
**HOXA-AS3**					
Atherosclerosis	Up	miR-455-5p	miR-455-5p /p27 Kip1	Proliferation, apoptosis	[144]
Endothelium inflammation			NF-κB		[145]
Pulmonary arterial hypertension	Up	miR-675-3p	miR-675-3p/PDE5A	Proliferation, viability, and migration	[146]

### HOXA-AS3 in diseases

In the investigation of atherosclerosis, the expression of HOXA-AS3 is positively associated with inflammatory atherosclerosis and interacts with NF-κB to induce endothelial inflammation [145]. Chi et al. have discovered that upregulation of HOXA-AS3 significantly promotes the progression of atherosclerosis by modulating the miR-455-5p/p27 Kip1 axis [144]. Additionally, HOXA-AS3 enhances the development of pulmonary arterial hypertension through the modulation of the miR-675-3p/PDE5 axis [146] (Table 2).

Moreover, multiple studies have demonstrated the tumorigenic role of HOXA-AS2/3 in various cancers, including CRC, gallbladder carcinoma (GBC), GC, HCC, pancreatic cancer (PC), malignant glioma, and others.

### HOXA-AS2 in cancers

In HCC, the downregulation of HOXA-AS2 inhibits cell proliferation and invasion, while promoting apoptosis. Conversely, high expression of HOXA-AS2 is associated with larger tumor size, advanced TNM stages, and shorter OS [147]. Similarly, in GC, HOXA-AS2 showed significant upregulation in cancerous tissues compared to noncancerous tissues [148]. Through its interaction with EZH2, HOXA-AS2 binds to the promoter region of PLK3, P21, and DDIT3, leading to their suppression via H3K27 trimethylation and contributing to the progression of GC [149]. Similar observations have been made in CRC [150]. Li et al. demonstrated that HOXA-AS2 is highly expressed in CRC tissues compared to normal tissues, and its expression can serve as a predictor of poor prognosis in CRC patients [151]. The association between HOXA-AS2 and EZH2 has also been confirmed in PC [152], OSCC [153], acute myeloid leukemia (AML) [154], and glioma [155]. Furthermore, the downregulation of HOXA-AS2 induces cell apoptosis, arrests the cell cycle in the G1 phase, and inhibits metastasis and invasion in GBC [156]. In glioma, HOXA-AS2 affects the expression of E2F8, E2F1, ATF3, and STAT1, promoting the proliferation of glioma stem cells (GSCs) and enhancing their aggressiveness [157]. In CC, HOXA-AS2 enhances proliferation and metastasis by activating the Notch signaling pathway [158]. Moreover, HOXA-AS2 enhances metastasis and invasion of NSCLC cells through the activation of IGF2 [159].

**Fig. 4:**
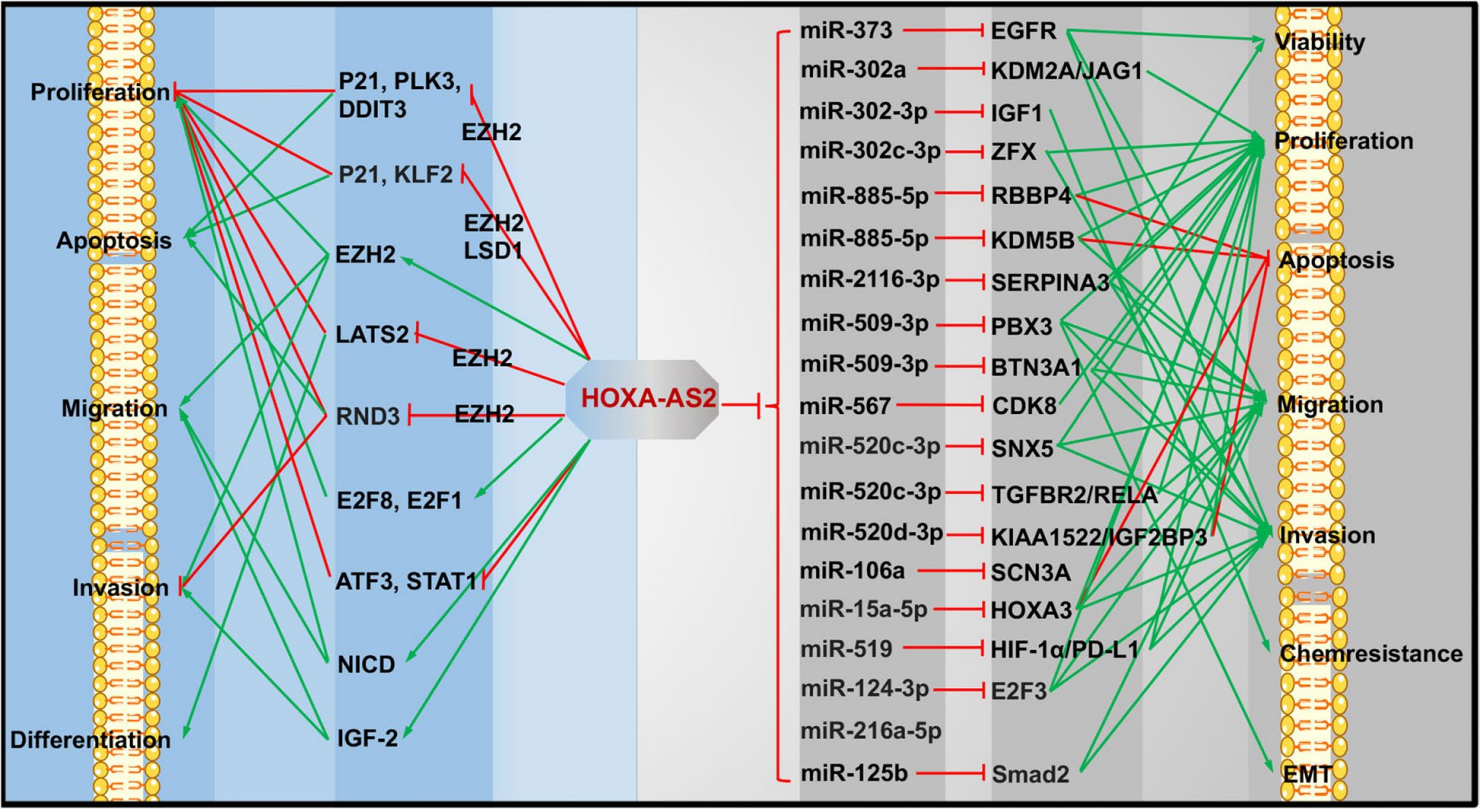
Role of HOXA- AS2 in tumor regulation

Accumulating evidence suggests that HOXA-AS2 acts as a miRNA sponge to facilitate tumor progression by regulating gene expression. For instance, in glioma, HOXA-AS2 interacts with miR-373 and miR-302a to modulate the expression of EGFR, KDM2A/JAG1, resulting in enhanced vasculogenic mimicry (VM) formation, cell viability, and metastasis [160], as well as immune tolerance [161]. Similarly, in GBM, HOXA-AS2 targets miR-2116-3p, miR-885-5p, and miR-302a-3p, thereby regulating the expression of SERPINA3, RBBP4, and IGF1, which influence GBM progression [162–164]. In PCa, HOXA-AS2 plays a role in the regulation of cell proliferation, migration, and EMT through the miR-885-5p/KDM5B and miR-509-3p/PBX3 axes [165, 166]. Furthermore, in OSCC, HOXA-AS2 promotes tumor progression by modulating CDK8 and SNX5, while suppressing miR-567 and miR-520c-3p, respectively [167, 168]. Moreover, HOXA-AS2 acts as a sponge for miR-520c-3p and miR-106a to regulate TGFBR2/RELA and SCN3A, respectively, thereby enhancing tumor progression in BC [169, 170]. Additionally, HOXA-AS2 is implicated in the development and progression of various other cancers, including CC [171], papillary thyroid cancer[172], type I endometrial carcinoma [173], NPC [174], osteosarcoma [175], NSCLC [176], bladder cancer (BCa) [177], and thoracic aortic aneurysm [178]. In these cases, HOXA-AS2 modulates different miRNA-related pathways, such as miR-509-3p/BTN3A1, miR-15a-5p/HOXA3, miRNA-302c-3p/ZFX, miR-519/HIF-1α/PD-L1, miR-124-3p/E2F3, miR-216a-5p, miR-125b/Smad2, and miR-520d-3p/KIAA1522/IGF2BP3, to promote tumor progression (Table 3 and Fig. 4).

**Table 3:** Role of HOXA- AS2 in tumor regulation

Cancers	Expression	Regulate genes	pathways	Functions	References
AML	Up	LATS2	Combine with EZH2	Proliferation, differentiation of AML cells	[154]
BC	Up	miR-520c-3p	miR-520c-3p/TGFBR2/RELA	Proliferation, metastasis	[169]
BC	Up	miR-106a	miR-106a/SCN3A		[170]
BCa	Up	miR-125b	miR-125b/Smad2	Migration, invasion and stemness	[177]
CC	Up	Notch intracellular domain (NICD)	Notch	Proliferation, migration	[158]
CC	Up	miR-509-3p	miR-509-3p/BTN3A1	Proliferation, migration and invasion	[171]
CRC	Up	P21, KLF2	Combine with EZH2 and LSD1	Proliferation, apoptosis	[150]
GBC	Up			Proliferation, EMT, migration and invasion	[156]
GBM	Up	miR-2116-3p	miR-2116-3p/SERPINA3	Viability, proliferation, migration, and invasion	[162]
GBM	Up	miR-885-5p	miR-885-5p/RBBP4	Proliferation, apoptosis	[163]
GBM	Up	miR-302-3p	miR-302-3p/IGF1	Temozolomide Resistance	[164]
GC	Up			Associated with clinicopathological features	[148]
GC	Up	P21, PLK3, and DDIT3	Combine with EZH2	Proliferation, apoptosis	[149]
Glioma	Up	RND3	Combine with EZH2	Proliferation, apoptosis, and invasion	[155]
Glioma	Up	miR-373	miR-373/EGFR	Viability, migration, invasion, and VM formation	[160]
Glioma	Up	miR-302a	miR-302a/KDM2A/JAG1	Proliferation, immune tolerance	[161]
GSCs	Up	E2F8, E2F1, ATF3, STAT1		Proliferation	[157]
HCC	Up			Proliferation	[147]
NPC	Up	miR-519	miR-519/HIF-1α/PD-L1	Proliferation, migration, and invasion	[174]
NSCLC	Up	IGF-2		Migration, invasion	[159]
NSCLC	Up	miR-216a-5p		Proliferation, migration, and invasion	[176]
OSCC	Up	EZH2		Proliferation, migration, and invasion	[153]
OSCC	Up	miR-567	miR-567/CDK8	Proliferation	[167]
OSCC	Up	miR-520c-3p	miR-520c-3p/SNX5	Proliferation, migration, and invasion	[168]
Osteosarcoma	Up	miR-124-3p	miR-124-3p/E2F3	Proliferation, migration, and invasion	[175]
Papillary thyroid cancer	Up	miR-15a-5p	miR-15a-5p/HOXA3	Proliferation, apoptosis, migration, and invasion	[172]
PC	Up		Combine with EZH2 and LSD1	Proliferation, apoptosis	[152]
PCa	Up	miR-885-5p	miR-885-5p/KDM5B	Proliferation, apoptosis, and migration	[165]
PCa	Up	miR-509-3p	miR-509-3p/PBX3	Proliferation, migration, invasion, and EMT	[166]
Type I endometrial carcinoma	Up	miR-302c-3p	miR-302c-3p/ZFX	Proliferation, invasion	[173]
Thoracic aortic aneurysm	Up	miR-520d-3p	miR-520d-3p/KIAA1522/IGF2BP3	Proliferation, apoptosis	[178]

*AML* Acute myeloid leukemia, *BC* Breast cancer, *BCa* bladder cancer, *CC* Cervical cancer, *CRC* colorectal cancer, *EOC* Epithelial ovarian cancer, *GBC* Gallbladder carcinoma, *GBM* Glioblastoma multiforme, *GC* Gastric cancer, *GSCs* Glioma stem cells, *HCC* Hepatocellular carcinoma, *NPC* Nasopharyngeal carcinoma, *NSCLC* Non‑small cell lung cancer, *OSCC* oral squamous cell carcinoma, *PC* Pancreatic cancer, *PCa* Prostate cancer

### HOXA-AS3 in cancers

**Fig. 5:**
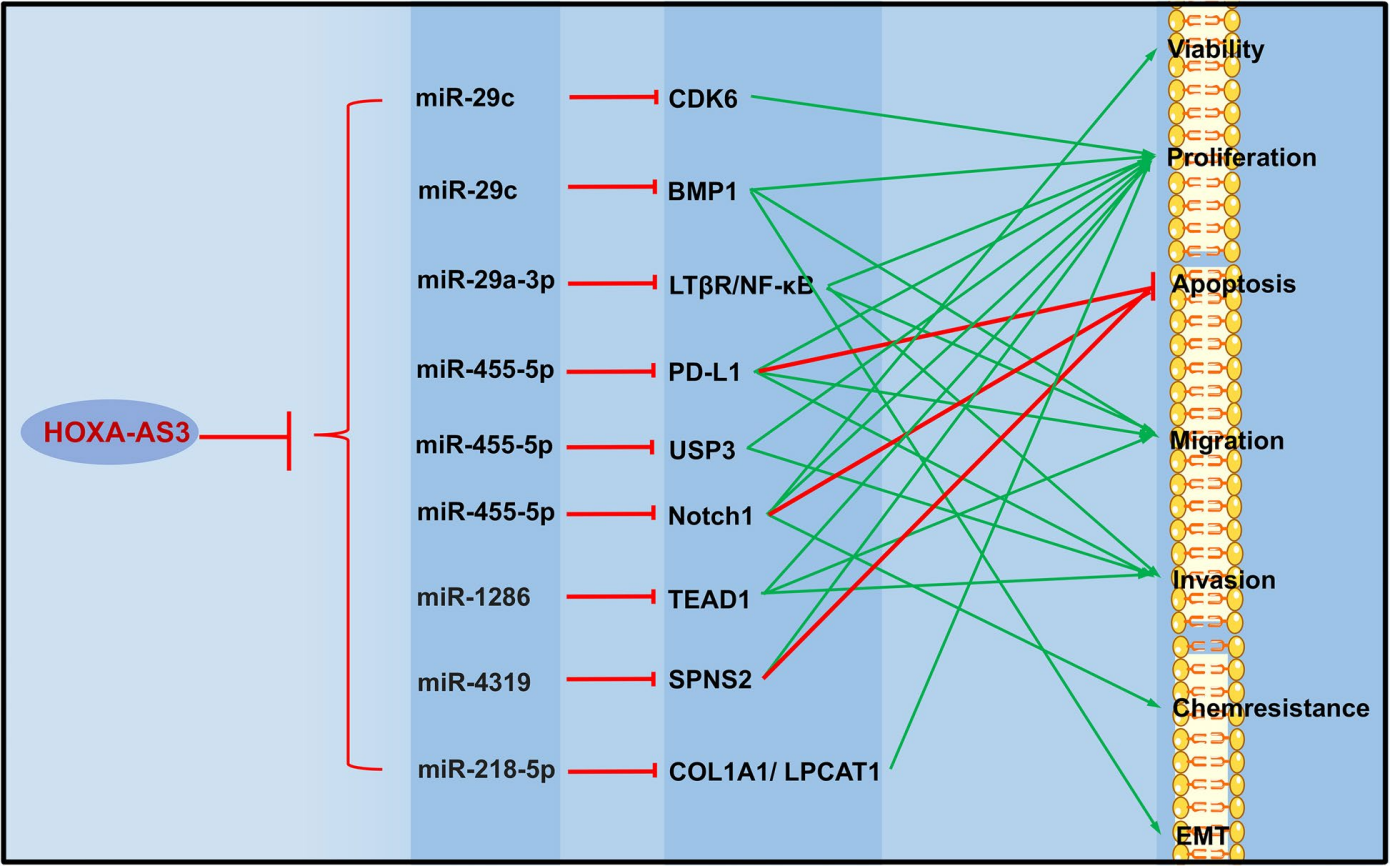
Role of HOXA- AS3 in tumor regulation

Kyung Jin Eoh et al. demonstrated in their study on EOC that a high expression of HOXA-AS3 is associated with shorter OS, and overexpression of HOXA-AS3 enhances the proliferation and metastasis of EOC cells [179]. In PC, HOXA-AS3 targets miR-29c to promote the expression of CDK6, thereby stimulating the growth of PC cells [180]. Meanwhile, the HOXA-AS3/miR-455-5p/PD-L1 [181] and miR-29c/ BMP1 [182] axis are implicated in the progression of HCC, as high expression of HOXA-AS3 leads to increased proliferation, metastasis, and invasion in HCC. In CC, HOXA-AS3 drives disease progression by modulating the activity of miR-29a-3p [183]. Furthermore, HOXA-AS3 interacts with HOXA3 to promote cisplatin resistance in NSCLC [80]. Simultaneously, HOXA-AS3 elevates the expression of TEAD1 through its competing endogenous RNA activity on miR-1286, ultimately inducing the proliferation and migration of HUVECs and EMT in osteosarcoma [184]. Moreover, HOXA-AS3 exerts regulatory control over miRNA-related pathways, including miR-455-5p/Notch1, miR-4319/SPNS2, miR-455-5p/USP3, miR-29a-3p/LTβR/NF-κB, miR-218-5p/COL1A1/LPCAT1, to promote progression in BCa [185], CRC [186], GBM [187], GC [188], and OSCC [189] (Table 4 and Fig. 5).

**Table 4:** Role of HOXA- AS3 in tumor regulation

Cancers	Expression	Regulate genes	pathways	Functions	References
BCa	Up	miR-455-5p	miR-455-5p/Notch1	Viability, proliferation, apoptosis, and chemoresistance	[185]
CC	Up	miR-29a-3p		Proliferation, migration, and invasion	[183]
CRC	Up	miR-4319	miR-4319/SPNS2	Proliferation, apoptosis	[186]
EOC	Up			Proliferation, metastasis	[179]
GBM	Up	miR-455-5p	miR-455-5p/USP3	Proliferation, invasion	[187]
GC	Up	miR-29a-3p	miR-29a-3p/LTβR/NF-κB	Proliferation, metastasis, and invasion	[188]
HCC	Up	miR-455-5p	miR-455-5p/PD-L1	Proliferation, apoptosis, metastasis, and invasion	[181]
HCC	Up	miR-29c	miR-29c/BMP1 MEK/ERK	Proliferation, metastasis, and EMT	[182]
NSCLC	Up	HOXA3		Cisplatin resistance	[80]
OSCC	Up	miR-218-5p	miR-218-5p/COL1A1/LPCAT1	Proliferation	[189]
Osteosarcoma	Up	miR-1286	miR-1286/TEAD1	Proliferation, migration, and invasion	[184]
PC	Up	miR-29c	miR-29c/CDK6	Proliferation	[180]

*BCa* bladder cancer, *CC* Cervical cancer, *CRC* colorectal cancer, *EOC* Epithelial ovarian cancer, *GBM* Glioblastoma multiforme, *GC* Gastric cancer, *HCC* Hepatocellular carcinoma, *OSCC* oral squamous cell carcinoma, *PC* Pancreatic cancer, *NSCLC* Non‑small cell lung cancer

These studies offer valuable insights into the involvement of HOXA-AS2/3 in the onset and progression of diverse diseases, thereby illuminating potential therapeutic targets and prognostic indicators.

### HOXA1-4 genes downstream: target genes and pathway modulation in cancers

#### Target genes

As a transcription factor, HOXA is pivotal in driving disease progression by targeting both the promoter and enhancer regions of genes. It exerts regulatory effects by binding to specific DNA sequences within these regions, thereby influencing the expression of downstream genes and contributing to the development and progression of a wide range of diseases.

The expression of HOXA1 has been found to be correlated with the transformation of VSMCs into macrophage-like cells. Mechanistic investigations have revealed that inhibiting HOXA1 leads to the suppression of transcriptional activation of NF-κB p65 and KLF4, thereby participating in the pathological manifestations of VSMCs [190]. Simultaneously, HOXA2 binds to a long-range enhancer of Hmx1, thereby regulating its expression. Hmx1 serves as a crucial transcription factor in the development of both the eye and ear [191].

In the context of cancer research, upregulation of HOXA1 has been observed in BC. Increased expression of HOXA1 has been shown to promote cell proliferation and enhance metastasis by directly binding to the promoter region of SMAD3, thus regulating its transcription [192]. Furthermore, HOXA1 has the ability to facilitate the enrichment of H3K4me1 and H3K27ac in the enhancer region of MEIS3, resulting in enhanced expression of MEIS3. MEIS3, in turn, regulates 3-phosphoinositide-dependent protein kinase 1 associated with the PI3K/AKT signaling pathway, thereby promoting migration and invasion in HCC [64]. Recent research has also uncovered a connection between transcriptional regulators of glycolytic metabolism and GBM progression. Specifically, HOXA3 activates the lysine-specific demethylase KDM6A, which then binds to binding sites on glycolytic genes and removes the histone modification H3K27me3. This process triggers aerobic glycolysis, promoting GBM progression [130]. Additionally, HOXA4 binds to the promoter region of ARHGAP25, exerting regulatory control over its transcriptional activity and influencing the growth, invasion, and migration of lung cancer cells [193]. HOXA4 also binds to the promoter of miR-138, which inhibits proliferation and gefitinib resistance in NSCLC [93].

### HOXAs as a signaling modulator

**Fig. 6:**
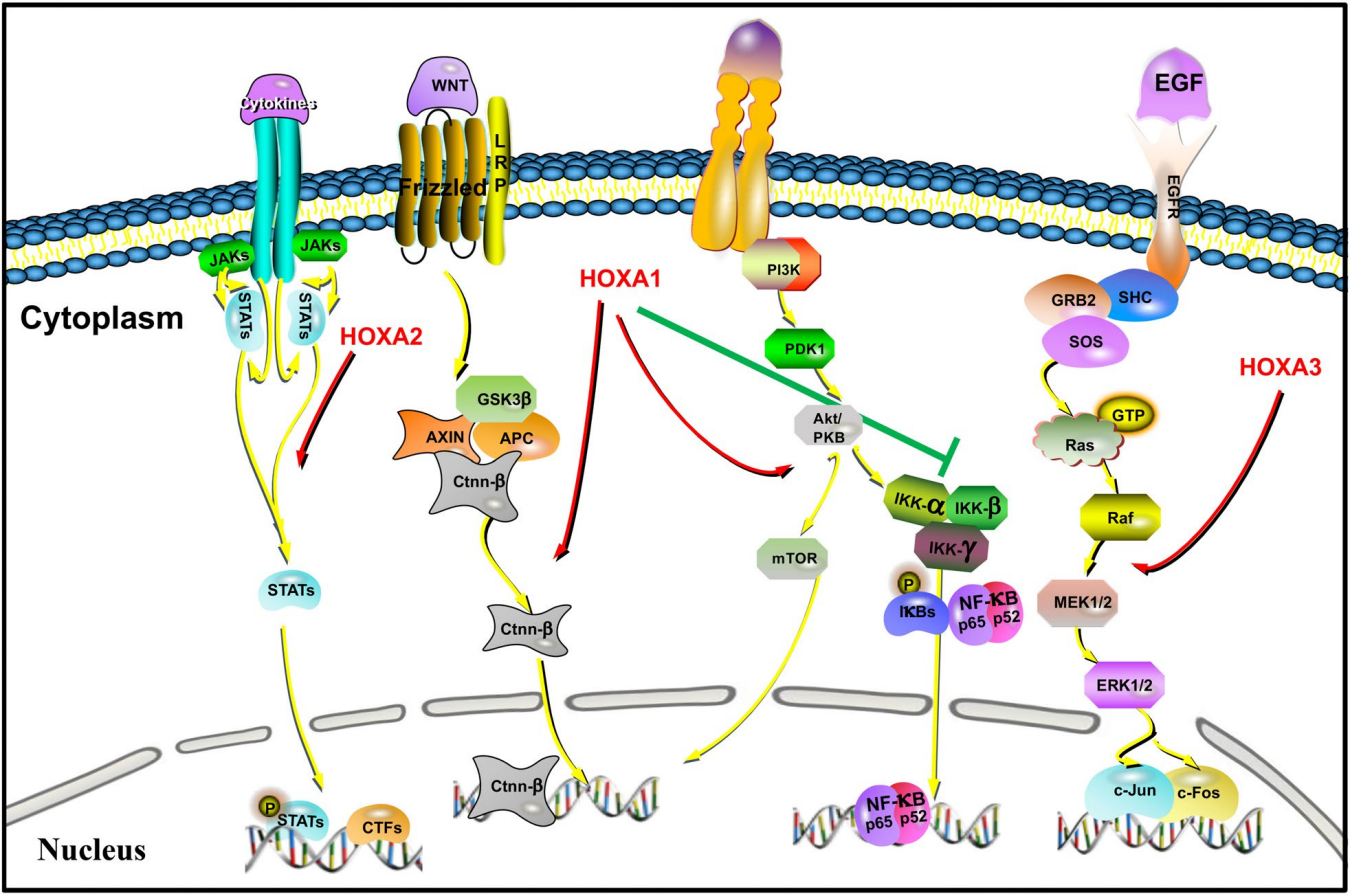
HOXA1-4 is involved in signaling pathways. These include the Wnt/β-catenin, JAK-STAT, MEK/ERK, and PI3K-AKT pathways

HOXAs play a role in regulating various signaling pathways, including the Wnt/β-catenin, JAK-STAT, and MEK/ERK pathways, among others, that are implicated in carcinogenesis and are crucial in multiple types of cancer (Fig. 6).

Utilizing bioinformatics tools such as GSEA and the Chinese Glioma Genome Atlas (CGGA), an analysis was conducted to investigate the impact of HOXA2 on glioma progression. Results showed that HOXA2 is highly expressed in tumor tissues compared to normal tissues. Overexpression of HOXA2 contributes to the activation of the JAK-STAT signaling pathway, the cell-adhesion-molecules (CAMS) pathway, focal adhesion, the cytosolic DNA sensing pathway, and natural killer cell-mediated cytotoxicity [194]. Additionally, the role of the Wnt/β-catenin pathway, known to be significant in oncogenesis and tumor development, has been explored in relation to HOXA1. Han et al. reported that miR-100 targets HOXA1, leading to increased invasion and migration abilities of NSCLC cells through the Wnt/β‐catenin pathway [70]. Similarly, the long non-coding RNA ZFPM2-AS1 acts as a competing endogenous RNA for miR-515, positively interacting with HOXA1 to activate the Wnt/β-catenin signaling pathway, promoting the development of retinoblastoma [85]. Furthermore, evidence suggests that HOXA1, functioning as a tumor suppressor gene, participates in the regulation of chemoresistance in small cell lung cancer (SCLC) through the NF-KB signaling pathway [195]. In OC, miR-99a targets HOXA1 to suppress OC proliferation by affecting the AKT/mTOR pathway [75]. Another notable pathway involving HOXA3 is the EGFR pathway. HOXA-AS2 enhances HOXA3 expression, thus activating the EGFR/Ras/Raf/MEK/ERK signaling pathway. This leads to increased glucocorticoid resistance by promoting proliferation and suppressing apoptosis in acute lymphoblastic leukemia cell lines [196].

### Cross-talk between HOXs

In various diseases, interactions and co-expression among HOXA family members have been demonstrated through bioinformatics data and in vitro and in vivo results. This network regulation of genes may contribute significantly to the development of diseases. For instance, in endometriosis, all members of the HOXA gene family (except for HOXA1) were significantly down-regulated in ectopic tissues compared to normal endometrium [30]. In tumors, analysis of TCGA data revealed that HOXA2, HOXA9, and HOXA10 are significant genes associated with PCa and are dysregulated in PCa, affecting the OS of PCa patients. Notably, the expression of HOXA2, HOXA9, and HOXA10 is related to immune infiltrates of immune cells [197]. Research conducted in KIRC indicated a significant increase in the expression of HOXA4 and HOXA13, while HOXA7 and HOXA11 were decreased. Furthermore, high expression of HOXA2, HOXA3, and HOXA13, along with low expression of HOXA7, is associated with poor OS in KIRC patients [198]. However, in laryngeal squamous cell cancer, expression of HOXA2 and HOXA4 was significantly downregulated, while HOXA7 and HOXA9-13 were upregulated [199]. Through the use of various public databases and bioinformatics analyses, it has been observed that 11 HOXAs (HOXA1, HOXA2, HOXA3, HOXA4, HOXA5, HOXA6, HOXA7, HOXA9, HOXA10, HOXA11, and HOXA13) show high expression in lower-grade glioma and GBM tissues [200]. Additionally, in early-stage CRC, high-frequency hypermethylation of HOXA2, HOXA5, and HOXA6 was identified as the main reason for their low expression in CRC [136].

In addition to co-expressions among members of the HOXA family, there are also co-expressions with other members of the HOX family. For instance, HOXA3 exhibited upregulation in ependymoma when combined with ARX, HOXA5, HOXA9, and HOXC6 [201]. Furthermore, mechanistic investigations have revealed that 2F1, HNF3α, KLF6, and SP3 have the ability to bind to promoter regions shared by all HOX genes, thereby regulating their expression collectively [202].

### HOXA1-4 genes potentiate disease treatment

Sclerostin (Sost), a secreted glycoprotein encoded by the mouse gene, plays a significant role in bone remodeling. Antibody therapy targeting sclerostin has been approved for the treatment of osteoporosis. In aged mice on a Western diet, knockouts of the Sost gene have been found to prevent aortic valve stenosis. Interestingly, the enhanced expression of HOXA1 was identified in valve interstitial cells of mice with the Sost gene knockout [203]. In studies on mouse embryos treated with phenytoin sodium, a known risk factor for cleft palate, a high expression of HOXA2 was observed in craniofacial tissues [204]. Furthermore, dihydroartemisinin, an extract derived from Artemisia apiacea, has demonstrated the ability to inhibit the differentiation of mesenchymal stem cells through the modulation of Runx2 and Col10a1 regulation by Pax6/HOXA2 [205].

In the investigation of HOXA function in tumor treatment, findings have revealed that the methylation of HOXA1 can be induced by HOTAIR, resulting in chemoresistance in SCLC via activation of the NF-κB pathway [195]. This mechanism has also been observed in cisplatin resistance in LUAD. Furthermore, a joint analysis of various databases has identified HOXA1, along with five other genes (BATF3, FOXA2, IRF5, SIX1, and ZBTB38), as a model for cisplatin resistance [206]. The combination of methylated HOXA1, CA19-9, and SST has been utilized for the diagnosis of stage I PC [207]. Additionally, the long non-coding RNA CCAT1 functions as a sponge for miR-218 to enhance gefitinib resistance in NSCLC by modulating HOXA1 [208]. KDM3A undergoes tyrosine phosphorylation, which in turn regulates the expression of HOXA1 to promote the growth of tamoxifen-resistant BC [209]. Radiotherapy is a commonly used treatment method for cancer patients, and in this context, HOXA1 has been identified as an independent predictor of tumor progression, exhibiting a positive correlation with the expression of genes that enhance radiation resistance, including EGFR, CDK6, and CAV1 [115]. Furthermore, when comparing imatinib mesylate-resistant chronic myeloid leukemia patients, the promoter hypermethylation of the HOXA4 gene was significantly higher in those who did not respond well to treatment [210]. Epigenetic silencing of HOXA4 is also associated with decreased sensitivity of chronic lymphocytic leukemia cells to treatment with fludarabine, ibrutinib, and idelalisib [211].

The broad expression and notable prognostic significance of HOXA1-4 genes have been observed across various human cancers. Consequently, targeting HOXA genes represents an appealing strategy for drug discovery, and extensive efforts have been devoted to developing therapies that selectively target these genes.

## Conclusion and prospective

In summary, HOXA1-4 genes play a crucial role in the regulation of various diseases and the progression of tumors. In common diseases, mutations, epigenetic modifications, and gene dysregulation are significant pathogenic factors contributing to conditions such as developmental defects in the head and face, cardiovascular diseases, viral infections, and neurological development, as well as inflammatory reactions. In cancer, HOXA1-4 genes can function as either oncogenes or tumor suppressors and interact with other members of the HOX family to regulate tumor proliferation, invasion, and metastasis through signaling pathways such as JAK-STAT, MEK/ERK, and Wnt/β-catenin. Additionally, HOXA-AS2/3 has been implicated in the progression of various diseases, including vascular disorders and inflammation. In cancer, HOXA-AS2/3 is frequently overexpressed in several types of cancer, such as CC, GBM, GC, NSCLC, and OSCC. The elevated expression of HOXA-AS2/3 is associated with poor prognosis, drug resistance, cancer metastasis, and lower survival rates. It primarily acts as a sponge for miRNAs, participating in the regulation of tumor development and progression. Furthermore, the expression of HOXA1-4 is also connected to chemoresistance and response to radiotherapy. Therefore, studying the mechanisms underlying the involvement of HOXA1-4 in diseases and tumors can establish a solid theoretical foundation for gene therapy. However, the application of HOXA1-4 in targeted therapy is currently in the preclinical or clinical trial stages, necessitating further research in this area.

## Data Availability

Not applicable.

## Abbreviations

ABDS: Athabascan Brain Dysgenesis Syndrome
AD: Alzheimer's disease
ADC: Adenocarcinoma
AML: Acute myeloid leukemia
BAV: Bicuspid aortic valve
BC: Breast cancer
BCa: Bladder cancer
CC: Cervical cancer
ccRCC: Clear cell renal cell carcinoma
CGGA: Chinese Glioma Genome Atlas
CHD: Congenital heart defects
circRNAs: Circular RNAs
CpG: Cytosine-phosphate-guanine
CRC: Colorectal cancer
EC: Endometrial cancer
EHC: Extrahepatic cholangiocarcinoma
EMT: Epithelial-mesenchymal transition
EOC: Epithelial ovarian cancer
ESCC: Esophageal squamous cell carcinoma
GBC: Gallbladder carcinoma
GBM: Glioblastoma multiforme
GC: Gastric cancer
GSCs: Glioma stem cells
GSEA: Gene Set Enrichment Analysis
GSVA: Gene Set Variation Analysis
HB: Hepatoblastoma
HCC: Hepatocellular carcinoma
HCV: Hepatitis C virus
HDCP: Hypertensive disorder complicating pregnancy
HF: Heart failure
HIBD: Hypoxic-ischemic brain damage
HNSCC: Head and neck squamous cell carcinoma
Hox: Homeobox
KIRC: Kidney renal clear cell carcinoma
LUAD: Lung adenocarcinoma
NCCs: Neural crest cells
NPC: Nasopharyngeal carcinoma
NSCLC: Non‑small cell lung cancer
NTDs: Neural tube defects
OC: Ovarian cancer
OS: Overall survival
OSCC: Oral squamous cell carcinoma
PC: Pancreatic cancer
PCa: Prostate cancer
PDAC: Pancreatic ductal adenocarcinoma
RB: Retinoblastoma
SCLC: Small cell lung cancer
SULEHS: Suleiman-El-Hattab syndrome
TC: Thyroid carcinoma
TCGA: The Cancer Genome Atlas
TEADs: Transcriptional enhancer activator domain
T‑LBL: T‑cell lymphoblastic lymphoma
UC: Ulcerative colitis
UCA1: Urothelial carcinoma associated 1

## References

[1] Bastida MF, Perez-Gomez R, Trofka A, Zhu J, Rada-Iglesias A, Sheth R, Stadler HS, Mackem S, Ros MA (2020). The formation of the thumb requires direct modulation of Gli3 transcription by Hoxa13. Proc Natl Acad Sci U S A.

[2] Melas M, Kautto EA, Franklin SJ, Mori M, McBride KL, Mosher TM, Pfau RB, Hernandez-Gonzalez ME, McGrath SD, Magrini VJ (2022). Long-read whole genome sequencing reveals HOXD13 alterations in synpolydactyly. Hum Mutat.

[3] Imai F, Adam M, Potter SS, Yoshida Y (2021). HoxD transcription factors define monosynaptic sensory-motor specificity in the developing spinal cord. Development.

[4] Wang Y, Han R, Xu Z, Sun X, Zhou C, Han B, He S, Cong H (2021). Upregulation of lncRNA147410.3 in the brain of mice with chronic toxoplasma infection promoted microglia apoptosis by regulating Hoxb3. Front Cell Neurosci.

[5] Yu J, Wang L, Pei P, Li X, Wu J, Qiu Z, Zhang J, Ao R, Wang S, Zhang T (2019). Reduced H3K27me3 leads to abnormal hox gene expression in neural tube defects. Epigenetics Chromatin.

[6] Zhong W, Peng H, Tian A, Wei Y, Li H, Tian J, Zhao X (2015). Expression of miRNA-1233 in placenta from patients with hypertensive disorder complicating pregnancy and its role in disease pathogenesis. Int J Clin Exp Med.

[7] Ptaschinski C, Hrycaj SM, Schaller MA, Wellik DM, Lukacs NW (2017). Hox5 paralogous genes modulate Th2 cell function during chronic allergic inflammation via regulation of Gata3. J Immunol.

[8] Cui Y, Zhang C, Li Y, Ma S, Cao W, Guan F (2021). HOXD1 functions as a novel tumor suppressor in kidney renal clear cell carcinoma. Cell Biol Int.

[9] Xu F, Shangguan X, Pan J, Yue Z, Shen K, Ji Y, Zhang W, Zhu Y, Sha J, Wang Y (2021). HOXD13 suppresses prostate cancer metastasis and BMP4-induced epithelial-mesenchymal transition by inhibiting SMAD1. Int J Cancer.

[10] Yan M, Yin X, Zhang L, Cui Y, Ma X (2022). High expression of HOXB3 predicts poor prognosis and correlates with tumor immunity in lung adenocarcinoma. Mol Biol Rep.

[11] Wang MQ, Yin QY, Chen YR, Zhu SL (2021). Diagnostic and prognostic value of HOXC family members in gastric cancer. Future Oncol.

[12] Wang L, Gao Y, Zhao X, Guo C, Wang X, Yang Y, Han C, Zhao L, Qin Y, Liu L (2020). HOXD3 was negatively regulated by YY1 recruiting HDAC1 to suppress progression of hepatocellular carcinoma cells via ITGA2 pathway. Cell Prolif.

[13] Wang L, Gao Y, Tong D, Wang X, Guo C, Guo B, Yang Y, Zhao L, Zhang J, Yang J (2021). MeCP2 drives hepatocellular carcinoma progression via enforcing HOXD3 promoter methylation and expression through the HB-EGF/EGFR pathway. Mol Oncol.

[14] Jing Y, Gao B, Han Z, Xia L, Xin S (2021). The protective effect of HOXA5 on carotid atherosclerosis occurs by modulating the vascular smooth muscle cell phenotype. Mol Cell Endocrinol.

[15] Lin J, Zhu H, Hong L, Tang W, Wang J, Hu H, Wu X, Chen Y, Liu G, Yang Q (2021). Coexpression of HOXA6 and PBX2 promotes metastasis in gastric cancer. Aging.

[16] Wu F, Wu S, Tong H, He W, Gou X (2019). HOXA6 inhibits cell proliferation and induces apoptosis by suppressing the PI3K/Akt signaling pathway in clear cell renal cell carcinoma. Int J Oncol.

[17] Engle EC (2007). Oculomotility disorders arising from disruptions in brainstem motor neuron development. Arch Neurol.

[18] Bosley TM, Salih MA, Alorainy IA, Oystreck DT, Nester M, Abu-Amero KK, Tischfield MA, Engle EC (2007). Clinical characterization of the HOXA1 syndrome BSAS variant. Neurology.

[19] Graeber CP, Hunter DG, Engle EC (2013). The genetic basis of incomitant strabismus: consolidation of the current knowledge of the genetic foundations of disease. Semin Ophthalmol.

[20] Bosley TM, Alorainy IA, Salih MA, Aldhalaan HM, Abu-Amero KK, Oystreck DT, Tischfield MA, Engle EC, Erickson RP (2008). The clinical spectrum of homozygous HOXA1 mutations. Am J Med Genet A.

[21] Qiao R, He Y, Pan B, Xiao S, Zhang X, Li J, Zhang Z, Hong Y, Xing Y, Ren J (2015). Understanding the molecular mechanisms of human microtia via a pig model of HOXA1 syndrome. Dis Model Mech.

[22] Roux M, Zaffran S (2016). Hox genes in cardiovascular development and diseases. J Dev Biol.

[23] Odelin G, Faucherre A, Marchese D, Pinard A, Jaouadi H, Le Scouarnec S, FranceGenRef C, Chiarelli R, Achouri Y, Faure E (2023). Variations in the poly-histidine repeat motif of HOXA1 contribute to bicuspid aortic valve in mouse and zebrafish. Nat Commun.

[24] Makki N, Capecchi MR (2012). Cardiovascular defects in a mouse model of HOXA1 syndrome. Hum Mol Genet.

[25] Ma Y, Wu Y, Chen J, Huang K, Ji B, Chen Z, Wang Q, Ma J, Shen S, Zhang J (2019). miR-10a-5p promotes chondrocyte apoptosis in osteoarthritis by targeting HOXA1. Mol Ther Nucleic Acids.

[26] Ding B, Xu S, Sun X, Gao J, Nie W, Xu H (2020). miR-18a-3p encourages apoptosis of chondrocyte in osteoarthritis via HOXA1 pathway. Curr Mol Pharmacol.

[27] Conciatori M, Stodgell CJ, Hyman SL, O’Bara M, Militerni R, Bravaccio C, Trillo S, Montecchi F, Schneider C, Melmed R (2004). Association between the HOXA1 A218G polymorphism and increased head circumference in patients with autism. Biol Psychiatry.

[28] Ingram JL, Stodgell CJ, Hyman SL, Figlewicz DA, Weitkamp LR, Rodier PM (2000). Discovery of allelic variants of HOXA1 and HOXB1: genetic susceptibility to autism spectrum disorders. Teratology.

[29] Devlin B, Bennett P, Cook EH, Dawson G, Gonen D, Grigorenko EL, McMahon W, Pauls D, Smith M, Spence MA (2002). No evidence for linkage of liability to autism to HOXA1 in a sample from the CPEA network. Am J Med Genet.

[30] Golestan Jahromi M, Aflatoonian R, Afsharian P, Aghajanpour S, Shahhoseini M, Aflatoonian A (2018). Altered expression of 3 paralogus HOX A-D clusters in endometriosis disease: a case-control study. Int J Reprod Biomed.

[31] Zhao J, He L, Yin L (2020). lncRNA NEAT1 binds to MiR-339-5p to increase HOXA1 and alleviate ischemic brain damage in neonatal mice. Mol Ther Nucleic Acids.

[32] Zhang X, Tang X, Pan L, Li Y, Li J, Li C (2022). Elevated lncRNA-UCA1 upregulates EZH2 to promote inflammatory response in sepsis-induced pneumonia via inhibiting HOXA1. Carcinogenesis.

[33] Thakuri BKC, Zhang J, Zhao J, Nguyen LN, Nguyen LNT, Khanal S, Cao D, Dang X, Schank M, Wu XY (2020). LncRNA HOTAIRM1 promotes MDSC expansion and suppressive functions through the HOXA1-miR124 axis during HCV infection. Sci Rep.

[34] Mukherjee A, Shrivastava S, Bhanja Chowdhury J, Ray R, Ray RB (2014). Transcriptional suppression of miR-181c by hepatitis C virus enhances homeobox A1 expression. J Virol.

[35] Nazarenko MS, Markov AV, Lebedev IN, Sleptsov AA, Frolov AV, Barbash OL, Puzyrev VP (2013). [DNA methylation profiling of the vascular tissues in the setting of atherosclerosis]. Mol Biol (Mosk).

[36] Zeybel M, Vatansever S, Hardy T, Sari AA, Cakalagaoglu F, Avci A, Zeybel GL, Karahuseyinoglu S, Bashton M, Mathers JC (2016). DNA methylation profiling identifies novel markers of progression in hepatitis B-related chronic liver disease. Clin Epigenetics.

[37] Sharp GC, Ho K, Davies A, Stergiakouli E, Humphries K, McArdle W, Sandy J, Davey Smith G, Lewis SJ, Relton CL (2017). Distinct DNA methylation profiles in subtypes of orofacial cleft. Clin Epigenetics.

[38] Chen Z, Zheng J, Hong H, Chen D, Deng L, Zhang X, Ling J, Wu L (2020). lncRNA HOTAIRM1 promotes osteogenesis of hDFSCs by epigenetically regulating HOXA2 via DNMT1 in vitro. J Cell Physiol.

[39] Zhang B, Sun T (2020). Transcription factors that regulate the pathogenesis of ulcerative colitis. Biomed Res Int.

[40] Xin H, Changchen W, Lei L, Meirong Y, Ye Z, Bo P (2019). The phenolyzer suite: prioritizing the candidate genes involved in microtia. Ann Otol Rhinol Laryngol.

[41] Si N, Meng X, Lu X, Liu Z, Qi Z, Wang L, Li C, Yang M, Zhang Y, Wang C (2020). Duplications involving the long range HMX1 enhancer are associated with human isolated bilateral concha-type microtia. J Transl Med.

[42] Deprez PM, Nichane MG, Lengele BG, Rezsohazy R, Nyssen-Behets C (2013). Molecular study of a Hoxa2 gain-of-function in chondrogenesis: a model of idiopathic proportionate short stature. Int J Mol Sci.

[43] Mace KA, Restivo TE, Rinn JL, Paquet AC, Chang HY, Young DM, Boudreau NJ (2009). HOXA3 modulates injury-induced mobilization and recruitment of bone marrow-derived cells. Stem Cells.

[44] Bertrand N, Roux M, Ryckebusch L, Niederreither K, Dolle P, Moon A, Capecchi M, Zaffran S (2011). Hox genes define distinct progenitor sub-domains within the second heart field. Dev Biol.

[45] Kameda Y, Nishimaki T, Takeichi M, Chisaka O (2002). Homeobox gene hoxa3 is essential for the formation of the carotid body in the mouse embryos. Dev Biol.

[46] Kameda Y, Watari-Goshima N, Nishimaki T, Chisaka O (2003). Disruption of the Hoxa3 homeobox gene results in anomalies of the carotid artery system and the arterial baroreceptors. Cell Tissue Res.

[47] Hernandez HG, Sandoval-Hernandez AG, Garrido-Gil P, Labandeira-Garcia JL, Zelaya MV, Bayon GF, Fernandez AF, Fraga MF, Arboleda G, Arboleda H (2018). Alzheimer’s disease DNA methylome of pyramidal layers in frontal cortex: laser-assisted microdissection study. Epigenomics.

[48] Turner DC, Gorski PP, Maasar MF, Seaborne RA, Baumert P, Brown AD, Kitchen MO, Erskine RM, Dos-Remedios I, Voisin S (2020). DNA methylation across the genome in aged human skeletal muscle tissue and muscle-derived cells: the role of HOX genes and physical activity. Sci Rep.

[49] Aonuma T, Moukette B, Kawaguchi S, Barupala NP, Sepulveda MN, Frick K, Tang Y, Guglin M, Raman SV, Cai C (2022). MiR-150 attenuates maladaptive cardiac remodeling mediated by long noncoding RNA MIAT and directly represses profibrotic Hoxa4. Circ Heart Fail.

[50] Lillvis JH, Erdman R, Schworer CM, Golden A, Derr K, Gatalica Z, Cox LA, Shen J, Vander Heide RS, Lenk GM (2011). Regional expression of HOXA4 along the aorta and its potential role in human abdominal aortic aneurysms. BMC Physiol.

[51] Kimura M, Horie T, Baba O, Ide Y, Tsuji S, Ruiz Rodriguez R, Watanabe T, Yamasaki T, Otani C, Xu S (2020). Homeobox A4 suppresses vascular remodeling by repressing YAP/TEAD transcriptional activity. EMBO Rep.

[52] He L, Tu HJ, He WF, Guo LL, Yu SX, Li J, Wu Q, Li J (2015). Lentiviral-mediated overexpression of homeobox A4 by human umbilical cord mesenchymal stem cells repairs full-thickness skin defects. Mol Med Rep.

[53] Fan X, Ping L, Sun H, Chen Y, Wang P, Liu T, Jiang R, Zhang X, Chen X (2020). Whole-exome sequencing of discordant monozygotic twin families for identification of candidate genes for microtia-atresia. Front Genet.

[54] Riedhammer KM, Burgemeister AL, Cantagrel V, Amiel J, Siquier-Pernet K, Boddaert N, Hertecant J, Kannouche PL, Pouvelle C, Htun S (2022). Suleiman-El-Hattab syndrome: a histone modification disorder caused by TASP1 deficiency. Hum Mol Genet.

[55] Li QS, Vasanthakumar A, Davis JW, Idler KB, Nho K, Waring JF, Saykin AJ (2021). Alzheimer’s disease neuroimaging I: association of peripheral blood DNA methylation level with Alzheimer’s disease progression. Clin Epigenetics.

[56] Zhao N, Teles F, Lu J, Koestler DC, Beck J, Boerwinkle E, Bressler J, Kelsey KT, Platz EA, Michaud DS (2023). Epigenome-wide association study using peripheral blood leukocytes identifies genomic regions associated with periodontal disease and edentulism in the atherosclerosis risk in communities study. J Clin Periodontol.

[57] Geller F, Feenstra B, Carstensen L, Pers TH, van Rooij IA, Korberg IB, Choudhry S, Karjalainen JM, Schnack TH, Hollegaard MV (2014). Genome-wide association analyses identify variants in developmental genes associated with hypospadias. Nat Genet.

[58] Chalasani NP, Porter K, Bhattacharya A, Book AJ, Neis BM, Xiong KM, Ramasubramanian TS, Edwards DKt, Chen I, Johnson S (2022). Validation of a novel multitarget blood test shows high sensitivity to detect early stage hepatocellular carcinoma. Clin Gastroenterol Hepatol.

[59] Chalasani NP, Ramasubramanian TS, Bhattacharya A, Olson MC, Edwards VD, Roberts LR, Kisiel JB, Reddy KR, Lidgard GP, Johnson SC (2021). A novel blood-based panel of methylated DNA and protein markers for detection of early-stage hepatocellular carcinoma. Clin Gastroenterol Hepatol.

[60] Kisiel JB, Dukek BA, Ghoz RVSRK, Yab HM, Berger TC, Taylor CK, Foote WR, Giama PH, Onyirioha NH (2019). Hepatocellular carcinoma detection by plasma methylated DNA: discovery, phase I pilot, and phase II clinical validation. Hepatology.

[61] Johnson AM, Dudek JM, Edwards DK, Myers TA, Joseph P, Laffin JJ, Bruinsma JJ (2021). Analytical validation of a novel multi-target blood-based test to detect hepatocellular carcinoma. Expert Rev Mol Diagn.

[62] Elfiky AM, Mohamed RH, Abd El-Hakam FE, Yassin MA, ElHefnawi M (2021). Targeted delivery of miR-218 via decorated hyperbranched polyamidoamine for liver cancer regression. Int J Pharm.

[63] Tao C, Sun H, Sang W, Li S (2019). miRNA-99a inhibits cell invasion and migration in liver cancer by directly targeting HOXA1. Oncol Lett.

[64] Zhang Y, Pan Q, Shao Z (2020). Tumor-suppressive role of microRNA-202-3p in hepatocellular carcinoma through the KDM3A/HOXA1/MEIS3 pathway. Front Cell Dev Biol.

[65] Liu G, Liu B, Liu X, Xie L, He J, Zhang J, Dong R, Ma D, Dong K, Ye M (2021). ARID1B/SUB1-activated lncRNA HOXA-AS2 drives the malignant behaviour of hepatoblastoma through regulation of HOXA3. J Cell Mol Med.

[66] Lyu P, Zhai Z, Hao Z, Zhang H, He J (2021). CircWHSC1 serves as an oncogene to promote hepatocellular carcinoma progression. Eur J Clin Invest.

[67] Liu LJ, Sun XY, Yang CX, Zou XY (2021). MiR-10a-5p restrains the aggressive phenotypes of ovarian cancer cells by inhibiting HOXA1. Kaohsiung J Med Sci.

[68] Zou D, Zhou Q, Wang D, Guan L, Yuan L, Li S (2016). The downregulation of microRNA-10b and its role in cervical cancer. Oncol Res.

[69] He C, Chen ZY, Li Y, Yang ZQ, Zeng F, Cui Y, He Y, Chen JB, Chen HQ (2019). miR-10b suppresses cell invasion and metastasis through targeting HOXA3 regulated by FAK/YAP signaling pathway in clear-cell renal cell carcinoma. BMC Nephrol.

[70] Han W, Ren X, Yang Y, Li H, Zhao L, Lin Z (2020). microRNA-100 functions as a tumor suppressor in non-small cell lung cancer via regulating epithelial-mesenchymal transition and wnt/beta-catenin by targeting HOXA1. Thorac Cancer.

[71] He W, Huang Y, Jiang CC, Zhu Y, Wang L, Zhang W, Huang W, Zhou T, Tang S (2020). miR-100 inhibits cell growth and proliferation by targeting HOXA1 in nasopharyngeal carcinoma. Onco Targets Ther.

[72] Zhang TJ, Xu ZJ, Wen XM, Gu Y, Ma JC, Yuan Q, Lin J, Zhou JD, Qian J (2022). SLIT2 promoter hypermethylation-mediated SLIT2-IT1/miR-218 repression drives leukemogenesis and predicts adverse prognosis in myelodysplastic neoplasm. Leukemia.

[73] Wang L, Sui M, Wang X (2019). miR–338–3p suppresses the malignancy of T–cell lymphoblastic lymphoma by downregulating HOXA3. Mol Med Rep.

[74] Men L, Nie D, Nie H (2019). microRNA–577 inhibits cell proliferation and invasion in non–small cell lung cancer by directly targeting homeobox A1. Mol Med Rep.

[75] Zhang L, Liu XL, Yuan Z, Cui J, Zhang H (2019). MiR-99a suppressed cell proliferation and invasion by directly targeting HOXA1 through regulation of the AKT/mTOR signaling pathway and EMT in ovarian cancer. Eur Rev Med Pharmacol Sci.

[76] Feng L, Wang R, Wang Y, Shen X, Shi Q, Lian M, Ma H, Fang J (2021). Silencing long non-coding RNA DLX6-AS1 or restoring microRNA-193b-3p enhances thyroid carcinoma cell autophagy and apoptosis via depressing HOXA1. J Cell Mol Med.

[77] Fang S, Shen Y, Chen B, Wu Y, Jia L, Li Y, Zhu Y, Yan Y, Li M, Chen R (2018). H3K27me3 induces multidrug resistance in small cell lung cancer by affecting HOXA1 DNA methylation via regulation of the lncRNA HOTAIR. Ann Transl Med.

[78] Li Q, Dong C, Cui J, Wang Y, Hong X (2018). Over-expressed lncRNA HOTAIRM1 promotes tumor growth and invasion through up-regulating HOXA1 and sequestering G9a/EZH2/Dnmts away from the HOXA1 gene in glioblastoma multiforme. J Exp Clin Cancer Res.

[79] Li X, Pang L, Yang Z, Liu J, Li W, Wang D (2019). LncRNA HOTAIRM1/HOXA1 axis promotes cell proliferation, migration and invasion in endometrial cancer. Onco Targets Ther.

[80] Lin S, Zhang R, An X, Li Z, Fang C, Pan B, Chen W, Xu G, Han W (2019). LncRNA HOXA-AS3 confers cisplatin resistance by interacting with HOXA3 in non-small-cell lung carcinoma cells. Oncogenesis.

[81] Zheng JJ, Du XJ, Wang HP, Zhou LY, Wang YJ, Zhang L, Xu H, Zhang J, Hu ZF (2019). Long non-coding RNA 00152 promotes cell proliferation in cervical cancer via regulating miR-216b-5p/HOXA1 axis. Eur Rev Med Pharmacol Sci.

[82] Lu H, Zhang Q, Sun Y, Wu D, Liu L (2020). LINC00689 induces gastric cancer progression via modulating the miR-338-3p/HOXA3 axis. J Gene Med.

[83] Zhang T, Su F, Lu YB, Ling XL, Dai HY, Yang TN, Zhang B, Zhao D, Hou XM (2022). MYC/MAX-activated LINC00958 promotes lung adenocarcinoma by oncogenic transcriptional reprogramming through HOXA1 activation. Front Oncol.

[84] Li J, Zeng T, Li W, Wu H, Sun C, Yang F, Yang M, Fu Z, Yin Y (2020). Long non-coding RNA SNHG1 activates HOXA1 expression via sponging miR-193a-5p in breast cancer progression. Aging.

[85] Lyv X, Wu F, Zhang H, Lu J, Wang L, Ma Y (2020). Long noncoding RNA ZFPM2-AS1 knockdown restrains the development of retinoblastoma by modulating the microRNA-515/HOXA1/Wnt/beta-catenin axis. Invest Ophthalmol Vis Sci.

[86] Chen L, Luo C, Xu Y, Hu J, Chen H (2023). Circ_0058063 regulates the development of esophageal cancer through miR-377-3p/HOXA1 axis. Anticancer Drugs.

[87] Feng C, Wang Q, Deng L, Peng N, Yang M, Wang X (2022). Hsa_circ_0074032 promotes prostate cancer progression through elevating homeobox A1 expression by serving as a microRNA-198 decoy. Andrologia.

[88] Zhang L, Ding F (2019). Hsa_circ_0008945 promoted breast cancer progression by targeting miR-338-3p. Onco Targets Ther.

[89] Mao Y, Zhang L, Li Y (2019). circEIF4G2 modulates the malignant features of cervical cancer via the miR–218/HOXA1 pathway. Mol Med Rep.

[90] Zhong W, Bao L, Yuan Y, Meng Y (2021). CircRASSF2 acts as a prognostic factor and promotes breast cancer progression by modulating miR-1205/HOXA1 axis. Bioengineered.

[91] Zhang Y, Li XJ, He RQ, Wang X, Zhang TT, Qin Y, Zhang R, Deng Y, Wang HL, Luo DZ (2018). Upregulation of HOXA1 promotes tumorigenesis and development of non–small cell lung cancer: a comprehensive investigation based on reverse transcription-quantitative polymerase chain reaction and bioinformatics analysis. Int J Oncol.

[92] Gan BL, He RQ, Zhang Y, Wei DM, Hu XH, Chen G (2018). Downregulation of HOXA3 in lung adenocarcinoma and its relevant molecular mechanism analysed by RT-qPCR, TCGA and in silico analysis. Int J Oncol.

[93] Tang X, Jiang J, Zhu J, He N, Tan J (2019). HOXA4-regulated miR-138 suppresses proliferation and gefitinib resistance in non-small cell lung cancer. Mol Genet Genomics.

[94] Gao L, He RQ, Huang ZG, Li GS, Zeng JH, Hou JY, Luo JY, Dang YW, Zhou HF, Kong JL (2022). Expression landscape and functional roles of HOXA4 and HOXA5 in lung adenocarcinoma. Int J Med Sci.

[95] Tsou JA, Galler JS, Siegmund KD, Laird PW, Turla S, Cozen W, Hagen JA, Koss MN, Laird-Offringa IA (2007). Identification of a panel of sensitive and specific DNA methylation markers for lung adenocarcinoma. Mol Cancer.

[96] Chung JH, Lee HJ, Kim BH, Cho NY, Kang GH (2011). DNA methylation profile during multistage progression of pulmonary adenocarcinomas. Virchows Arch.

[97] Zhao N, Ruan M, Koestler DC, Lu J, Marsit CJ, Kelsey KT, Platz EA, Michaud DS (2022). Epigenome-wide scan identifies differentially methylated regions for lung cancer using pre-diagnostic peripheral blood. Epigenetics.

[98] Zhao F, Tian H, Liu X, Guan Y, Zhu Y, Ren P, Zhang J, Dong Y, Fu L (2022). Homeobox A1 facilitates immune escape and alleviates oxidative stress in lung adenocarcinoma. Oxid Med Cell Longev.

[99] Makiyama K, Hamada J, Takada M, Murakawa K, Takahashi Y, Tada M, Tamoto E, Shindo G, Matsunaga A, Teramoto K (2005). Aberrant expression of HOX genes in human invasive breast carcinoma. Oncol Rep.

[100] Belpaire M, Ewbank B, Taminiau A, Bridoux L, Deneyer N, Marchese D, Lima-Mendez G, Baurain JF, Geerts D, Rezsohazy R (2021). HOXA1 is an antagonist of ERalpha in breast Cancer. Front Oncol.

[101] Li SY, Wu HC, Mai HF, Zhen JX, Li GS, Chen SJ (2019). Microarray-based analysis of whole-genome DNA methylation profiling in early detection of breast cancer. J Cell Biochem.

[102] Liu J, Liu J, Lu X (2019). HOXA1 upregulation is associated with poor prognosis and tumor progression in breast cancer. Exp Ther Med.

[103] Wang H, Liu G, Shen D, Ye H, Huang J, Jiao L, Sun Y (2015). HOXA1 enhances the cell proliferation, invasion and metastasis of prostate cancer cells. Oncol Rep.

[104] Gao P, Xia JH, Sipeky C, Dong XM, Zhang Q, Yang Y, Zhang P, Cruz SP, Zhang K, Zhu J (2018). Biology and clinical implications of the 19q13 aggressive prostate cancer susceptibility locus. Cell.

[105] Emami NC, Kachuri L, Meyers TJ, Das R, Hoffman JD, Hoffmann TJ, Hu D, Shan J, Feng FY, Ziv E (2019). Association of imputed prostate cancer transcriptome with disease risk reveals novel mechanisms. Nat Commun.

[106] Yuan C, Zhu X, Han Y, Song C, Liu C, Lu S, Zhang M, Yu F, Peng Z, Zhou C (2016). Elevated HOXA1 expression correlates with accelerated tumor cell proliferation and poor prognosis in gastric cancer partly via cyclin D1. J Exp Clin Cancer Res.

[107] Kang GH, Lee S, Cho NY, Gandamihardja T, Long TI, Weisenberger DJ, Campan M, Laird PW (2008). DNA methylation profiles of gastric carcinoma characterized by quantitative DNA methylation analysis. Lab Invest.

[108] Degl’Innocenti R, Castiglione D, Buccoliero F, Bechi AM, Taddei P, Freschi GL, Taddei G (2007). Quantitative expression of the homeobox and integrin genes in human gastric carcinoma. Int J Mol Med.

[109] Eoh KJ, Kim HJ, Lee JY, Nam EJ, Kim S, Kim SW, Kim YT (2017). Dysregulated expression of homeobox family genes may influence survival outcomes of patients with epithelial ovarian cancer: analysis of data from the Cancer Genome Atlas. Oncotarget.

[110] Miller KR, Patel JN, Zhang Q, Norris EJ, Symanowski J, Michener C, Sehouli J, Braicu I, Destephanis DD, Sutker AP (2018). HOXA4/HOXB3 gene expression signature as a biomarker of recurrence in patients with high-grade serous ovarian cancer following primary cytoreductive surgery and first-line adjuvant chemotherapy. Gynecol Oncol.

[111] Klausen C, Leung PC, Auersperg N (2009). Cell motility and spreading are suppressed by HOXA4 in ovarian cancer cells: possible involvement of beta1 integrin. Mol Cancer Res.

[112] Ota T, Klausen C, Salamanca MC, Woo HL, Leung PC, Auersperg N (2009). Expression and function of HOXA genes in normal and neoplastic ovarian epithelial cells. Differentiation.

[113] Zhao Q, Zhang Y, Zhang X, Sun Y, Lin Z (2020). Mining of gene modules and identification of key genes in head and neck squamous cell carcinoma based on gene co-expression network analysis. Medicine (Baltimore).

[114] Li H, Wang X, Zhang M, Wang M, Zhang J, Ma S (2020). Identification of HOXA1 as a novel biomarker in prognosis of head and neck squamous cell carcinoma. Front Mol Biosci.

[115] He L, Liang M, Guo W, Liu J, Yu Y (2022). HOXA1 is a radioresistance marker in multiple cancer types. Front Oncol.

[116] Guo H, Li C, Su X, Huang X (2021). A five-mRNA expression signature to predict survival in oral squamous cell carcinoma by integrated bioinformatic analyses. Genet Test Mol Biomarkers.

[117] Bitu CC, Destro MF, Carrera M, da Silva SD, Graner E, Kowalski LP, Soares FA, Coletta RD (2012). HOXA1 is overexpressed in oral squamous cell carcinomas and its expression is correlated with poor prognosis. BMC Cancer.

[118] Rivera C, Gonzalez-Arriagada WA, Loyola-Brambilla M, de Almeida OP, Coletta RD, Venegas B (2014). Clinicopathological and immunohistochemical evaluation of oral and oropharyngeal squamous cell carcinoma in Chilean population. Int J Clin Exp Pathol.

[119] Padam KSR, Morgan R, Hunter K, Chakrabarty S, Kumar NAN, Radhakrishnan R (2022). Identification of HOX signatures contributing to oral cancer phenotype. Sci Rep.

[120] Majumder S, Taylor WR, Foote PH, Berger CK, Wu CW, Mahoney DW, Bamlet WR, Burger KN, Postier N, de la Fuente J (2021). High detection rates of pancreatic cancer across stages by plasma assay of novel methylated DNA markers and CA19-9. Clin Cancer Res.

[121] Katsuta E, Huyser M, Yan L, Takabe K (2021). A prognostic score based on long-term survivor unique transcriptomic signatures predicts patient survival in pancreatic ductal adenocarcinoma. Am J Cancer Res.

[122] Sharma G, Agarwal SM (2014). Identification of critical microRNA gene targets in cervical cancer using network properties. Microrna.

[123] Ge F, Tie W, Zhang J, Zhu Y, Fan Y (2021). Expression of the HOXA gene family and its relationship to prognosis and immune infiltrates in cervical cancer. J Clin Lab Anal.

[124] Shim C, Zhang W, Rhee CH, Lee JH (1998). Profiling of differentially expressed genes in human primary cervical cancer by complementary DNA expression array. Clin Cancer Res.

[125] Zhang Z, Peng J, Li B, Wang Z, Wang H, Wang Y, Hong L (2023). HOXA1 promotes aerobic glycolysis and cancer progression in cervical cancer. Cell Signal.

[126] Qin S, Liao Y, Du Q, Wang W, Huang J, Liu P, Shang C, Liu T, Xia M, Yao S (2020). DSG2 expression is correlated with poor prognosis and promotes early-stage cervical cancer. Cancer Cell Int.

[127] Wang YX, Zhang CQ, Han F (2020). [Effects of HOXA1 gene antisense oligonucleotides on growth of esophageal cancer cells]. Sichuan Da Xue Xue Bao Yi Xue Ban.

[128] Zheng ZQ, Yuan GQ, Zhang GG, Nie QQ, Wang Z (2023). Development and validation of a predictive model in diagnosis and prognosis of primary glioblastoma patients based on Homeobox A family. Discov Oncol.

[129] Xia H, Liu Y, Wang Z, Zhang W, Qi M, Qi B, Jiang X (2020). Long noncoding RNA HOTAIRM1 maintains tumorigenicity of glioblastoma stem-like cells through regulation of HOX gene expression. Neurotherapeutics.

[130] Yang R, Zhang G, Dong Z, Wang S, Li Y, Lian F, Liu X, Li H, Wei X, Cui H (2023). Homeobox A3 and KDM6A cooperate in transcriptional control of aerobic glycolysis and glioblastoma progression. Neuro Oncol.

[131] Qin G, Hu B, Li X, Li R, Meng Y, Wang Y, Zou D, Wei F (2020). Identification of Key differentially expressed transcription factors in glioblastoma. J Oncol.

[132] Yu Z, Liu Z, Lian X, Cheng X, Liu B, Zhang B, Wang H, Wang J, Li A, Ren Z (2022). High expression of HOXA4 in patients with glioma indicates unfavorable clinical outcomes. Cell Cycle.

[133] Wu ZH, Zhou T, Sun HY (2020). DNA methylation-based diagnostic and prognostic biomarkers of nasopharyngeal carcinoma patients. Medicine (Baltimore).

[134] Kim BH, Cho NY, Shin SH, Kwon HJ, Jang JJ, Kang GH (2009). CpG island hypermethylation and repetitive DNA hypomethylation in premalignant lesion of extrahepatic cholangiocarcinoma. Virchows Arch.

[135] Baharudin R, Ishak M, Muhamad Yusof A, Saidin S, Syafruddin SE, Wan Mohamad Nazarie WF, Lee LH, Ab Mutalib NS (2022). Epigenome-wide DNA methylation profiling in colorectal cancer and normal adjacent colon using infinium human methylation 450K. Diagnostics (Basel).

[136] Li D, Bai Y, Feng Z, Li W, Yang C, Guo Y, Lin C, Zhang Y, He Q, Hu G (2019). Study of promoter methylation patterns of HOXA2, HOXA5, and HOXA6 and its clinicopathological characteristics in colorectal cancer. Front Oncol.

[137] Li X, Yu HM (2020). Overexpression of HOXA-AS2 inhibits inflammation and apoptosis in podocytes via sponging miRNA-302b-3p to upregulate TIMP3. Eur Rev Med Pharmacol Sci.

[138] Gao K, Lv A, Zhang Q, Li Y, Yue Z, Xu S (2023). Long noncoding RNA HOXA-AS2 ameliorates chronic intermittent hypoxia-induced lung inflammation by regulating miR-17-5p/tipe2 axis. Allergol Immunopathol (Madr).

[139] Fan TT, Liu YX, Wang XC, Xu BL, Chen ZC, Lu HA, Zhang M (2020). LncRNA HOXA-AS2 accelerates the proliferation and migration and inhibits the apoptosis of vascular smooth muscle cells by absorbing miRNA-877-3p. Eur Rev Med Pharmacol Sci.

[140] Zhu X, Liu Y, Yu J, Du J, Guo R, Feng Y, Zhong G, Jiang Y, Lin J (2019). LncRNA HOXA-AS2 represses endothelium inflammation by regulating the activity of NF-kappaB signaling. Atherosclerosis.

[141] Song L, Li J, Sun Z (2023). Knocking down lncRNA HOXA-AS2 mitigates the progression of epilepsy via regulation of the miR-372-3p/STAT3 axis. Turk Neurosurg.

[142] Yang X, Zhang Y, Chen Y, He X, Qian Y, Xu S, Gao C, Mo C, Chen S, Xiao Q (2021). LncRNA HOXA-AS2 regulates microglial polarization via recruitment of PRC2 and epigenetic modification of PGC-1alpha expression. J Neuroinflammation.

[143] Wu W, Jing Y, Xu Q, Hao J, Yu X (2021). Upregulated level of lncRNA HOXA-AS2 in peripheral blood of systemic lupus erythematosus patients aggravates disease progression via ERK pathway. Minerva Med.

[144] Chi K, Zhang J, Sun H, Liu Y, Li Y, Yuan T, Zhang F (2020). Knockdown of lncRNA HOXA-AS3 suppresses the progression of atherosclerosis via sponging miR-455-5p. Drug Des Devel Ther.

[145] Zhu X, Chen D, Liu Y, Yu J, Qiao L, Lin S, Chen D, Zhong G, Lu X, Wang Y (2019). Long noncoding RNA HOXA-AS3 integrates NF-kappaB signaling to regulate endothelium inflammation. Mol Cell Biol.

[146] Li ZK, Gao LF, Zhu XA, Xiang DK (2021). LncRNA HOXA-AS3 promotes the progression of pulmonary arterial hypertension through mediation of miR-675-3p/PDE5A axis. Biochem Genet.

[147] Wang F, Yang H, Deng Z, Su Y, Fang Q, Yin Z (2016). HOX antisense lincRNA HOXA-AS2 promotes tumorigenesis of hepatocellular carcinoma. Cell Physiol Biochem.

[148] Rajabi A, Riahi A, Shirabadi-Arani H, Moaddab Y, Haghi M, Safaralizadeh R (2022). Overexpression of HOXA-AS2 lncRNA in patients with gastric cancer and its association with helicobacter pylori infection. J Gastrointest Cancer.

[149] Xie M, Sun M, Zhu YN, Xia R, Liu YW, Ding J, Ma HW, He XZ, Zhang ZH, Liu ZJ (2015). Long noncoding RNA HOXA-AS2 promotes gastric cancer proliferation by epigenetically silencing P21/PLK3/DDIT3 expression. Oncotarget.

[150] Ding J, Xie M, Lian Y, Zhu Y, Peng P, Wang J, Wang L, Wang K (2017). Long noncoding RNA HOXA-AS2 represses P21 and KLF2 expression transcription by binding with EZH2, LSD1 in colorectal cancer. Oncogenesis.

[151] Li Q, Dai Y, Wang F, Hou S (2016). Differentially expressed long non-coding RNAs and the prognostic potential in colorectal cancer. Neoplasma.

[152] Lian Y, Li Z, Fan Y, Huang Q, Chen J, Liu W, Xiao C, Xu H (2017). The lncRNA-HOXA-AS2/EZH2/LSD1 oncogene complex promotes cell proliferation in pancreatic cancer. Am J Transl Res.

[153] Zhao Z, Xing Y, Yang F, Zhao Z, Shen Y, Song J, Jing S (2021). LncRNA HOXA-AS2 promotes oral squamous cell proliferation, migration, and invasion via upregulating EZH2 as an oncogene. Technol Cancer Res Treat.

[154] Feng Y, Hu S, Li L, Peng X, Chen F (2020). Long noncoding RNA HOXA-AS2 functions as an oncogene by binding to EZH2 and suppressing LATS2 in acute myeloid leukemia (AML). Cell Death Dis.

[155] Wu L, Zhu X, Song Z, Chen D, Guo M, Liang J, Ding D, Wang W, Yan D (2019). Long non-coding RNA HOXA-AS2 enhances the malignant biological behaviors in glioma by epigenetically regulating RND3 expression. Onco Targets Ther.

[156] Zhang P, Cao P, Zhu X, Pan M, Zhong K, He R, Li Y, Jiao X, Gao Y (2017). Upregulation of long non-coding RNA HOXA-AS2 promotes proliferation and induces epithelial-mesenchymal transition in gallbladder carcinoma. Oncotarget.

[157] Le Boiteux E, Guichet PO, Masliantsev K, Montibus B, Vaurs-Barriere C, Gonthier-Gueret C, Chautard E, Verrelle P, Karayan-Tapon L, Fogli A (2022). The long non-coding RNA HOXA-AS2 promotes proliferation of glioma stem cells and modulates their inflammation pathway mainly through post-transcriptional regulation. Int J Mol Sci.

[158] Wu Q, Lu S, Zhang L, Zhao L (2021). LncRNA HOXA-AS2 activates the notch pathway to promote cervical cancer cell proliferation and migration. Reprod Sci.

[159] Zheng FX, Wang XQ, Zheng WX, Zhao J (2019). Long noncoding RNA HOXA-AS2 promotes cell migration and invasion via upregulating IGF-2 in non-small cell lung cancer as an oncogene. Eur Rev Med Pharmacol Sci.

[160] Gao Y, Yu H, Liu Y, Liu X, Zheng J, Ma J, Gong W, Chen J, Zhao L, Tian Y (2018). Long non-coding RNA HOXA-AS2 regulates malignant glioma behaviors and vasculogenic mimicry formation via the MiR-373/EGFR axis. Cell Physiol Biochem.

[161] Zhong C, Tao B, Li X, Xiang W, Peng L, Peng T, Chen L, Xia X, You J, Yang X (2022). HOXA-AS2 contributes to regulatory T cell proliferation and immune tolerance in glioma through the miR-302a/KDM2A/JAG1 axis. Cell Death Dis.

[162] Sun J, Wang L (2022). HOXA-AS2 enhances GBM cell malignancy by suppressing mir-2116-3p thereby upregulating SERPINA3. BMC Cancer.

[163] Shou J, Gao H, Cheng S, Wang B, Guan H (2021). LncRNA HOXA-AS2 promotes glioblastoma carcinogenesis by targeting miR-885-5p/RBBP4 axis. Cancer Cell Int.

[164] Lin L, Lin D, Jin L, Wang J, Lin Z, Zhang S, Lin G (2022). LncRNA HOXA-AS2 promotes temozolomide resistance in glioblastoma by regulated miR-302a-3p/IGF1 axis. Genet Res (Camb).

[165] Yang Z, Zhang F, Cai K, Xu J (2023). LncRNA HOXA-AS2 facilitates prostate cancer progression by inhibiting mir-885-5p to upregulate KDM5B. Kidney Blood Press Res.

[166] Xiao S, Song B (2020). LncRNA HOXA-AS2 promotes the progression of prostate cancer via targeting miR-509-3p/PBX3 axis. Biosci Rep.

[167] Chen R, Wang X, Zhou S, Zeng Z (2021). LncRNA HOXA-AS2 promotes tumor progression by suppressing miR-567 expression in oral squamous cell carcinoma. Cancer Manag Res.

[168] Li Z (2022). Overexpression of lncRNA HOXA-AS2 promotes the progression of oral squamous cell carcinoma by mediating SNX5 expression. BMC Mol Cell Biol.

[169] Fang Y, Wang J, Wu F, Song Y, Zhao S, Zhang Q (2017). Long non-coding RNA HOXA-AS2 promotes proliferation and invasion of breast cancer by acting as a miR-520c-3p sponge. Oncotarget.

[170] Wu J, Li M, Zhang Y (2019). Long noncoding RNA HOXA-AS2 regulates the expression of SCN3A by sponging miR-106a in breast cancer. J Cell Biochem.

[171] Chen R, He P (2021). Long noncoding RNA HOXA-AS2 accelerates cervical cancer by the miR-509-3p/BTN3A1 axis. J Pharm Pharmacol.

[172] Jiang L, Wu Z, Meng X, Chu X, Huang H, Xu C (2019). LncRNA HOXA-AS2 facilitates tumorigenesis and progression of papillary thyroid cancer by modulating the miR-15a-5p/HOXA3 axis. Hum Gene Ther.

[173] Song N, Zhang Y, Kong F, Yang H, Ma X (2020). HOXA-AS2 promotes type I endometrial carcinoma via miRNA-302c-3p-mediated regulation of ZFX. Cancer Cell Int.

[174] Wang S, You H, Yu S (2020). Long non-coding RNA HOXA-AS2 promotes the expression levels of hypoxia-inducible factor-1alpha and programmed death-ligand 1, and regulates nasopharyngeal carcinoma progression via miR-519. Oncol Lett.

[175] Wang L, Wang L, Zhang X (2019). Knockdown of lncRNA HOXA-AS2 inhibits viability, migration and invasion of osteosarcoma cells by miR-124-3p/E2F3. Onco Targets Ther.

[176] Cui TJ, Lin GS, Dai YM, Zheng JP, Chen Z, Chen Q, Zheng Y, Lin X (2019). LncRNA HOXA-AS2 regulates microRNA-216a-5p to promote malignant progression of non-small cell lung cancer. Eur Rev Med Pharmacol Sci.

[177] Wang F, Wu D, Chen J, Chen S, He F, Fu H, Wu Q, Liu S, Li X, Wang W (2019). Long non-coding RNA HOXA-AS2 promotes the migration, invasion and stemness of bladder cancer via regulating miR-125b/Smad2 axis. Exp Cell Res.

[178] Ou M, Chu Y, Zhang Q, Zhao H, Song Q (2022). HOXA cluster antisense RNA 2 elevates KIAA1522 expression through microRNA-520d-3p and insulin like growth factor 2 mRNA binding protein 3 to promote the growth of vascular smooth muscle cells in thoracic aortic aneurysm. ESC Heart Fail.

[179] Eoh KJ, Lee DW, Nam EJ, Kim JI, Moon H, Kim SW, Kim YT (2023). HOXA–AS3 induces tumor progression through the epithelial–mesenchymal transition pathway in epithelial ovarian cancer. Oncol Rep.

[180] Zhang X, Zhu H, Qu X, Yu Z, Zhang J (2021). Suppressing lncRNA HOXA-AS3 by CRISPR-dCas9 inhibits pancreatic cancer development. J Cancer.

[181] Zeng C, Ye S, Chen Y, Zhang Q, Luo Y, Gai L, Luo B (2021). HOXA-AS3 promotes proliferation and migration of hepatocellular carcinoma cells via the miR-455-5p/PD-L1 axis. J Immunol Res.

[182] Tong Y, Wang M, Dai Y, Bao D, Zhang J, Pan H (2019). LncRNA HOXA-AS3 sponges miR-29c to facilitate cell proliferation, metastasis, and EMT process and activate the MEK/ERK signaling pathway in hepatocellular carcinoma. Hum Gene Ther Clin Dev.

[183] Xu H, Tang Y, He C, Tian Y, Ni R (2022). Prognostic value of lncRNA HOXA-AS3 in cervical cancer by targeting miR-29a-3p and its regulatory effect on tumor progression. J Obstet Gynaecol Res.

[184] Xiao X, Liu M, Xie S, Liu C, Huang X, Huang X (2023). Long non-coding HOXA-AS3 contributes to osteosarcoma progression through the miR-1286/TEAD1 axis. J Orthop Surg Res.

[185] Chen D, Xie S, Wu Y, Cui Y, Cai Y, Lan L, Yang H, Chen J, Chen W (2020). Reduction of bladder cancer chemosensitivity induced by the effect of HOXA-AS3 as a ceRNA for mir-455-5p that upregulates Notch1. Front Oncol.

[186] Jiang Y, Yu XY, Sun HX, Gu XY, Geng JS (2021). Long non-coding RNA HOXA-AS3 facilitates the malignancy in colorectal cancer by miR-4319/SPNS2 axis. J Physiol Biochem.

[187] Chen W, Li Q, Zhang G, Wang H, Zhu Z, Chen L (2020). LncRNA HOXA-AS3 promotes the malignancy of glioblastoma through regulating miR-455-5p/USP3 axis. J Cell Mol Med.

[188] Qu F, Zhu B, Hu YL, Mao QS, Feng Y (2021). LncRNA HOXA-AS3 promotes gastric cancer progression by regulating miR-29a-3p/LTbetaR and activating NF-kappaB signaling. Cancer Cell Int.

[189] Zhao Y, Yao R (2021). Long non-coding RNA HOXA-AS3 promotes cell proliferation of oral squamous cell carcinoma through sponging microRNA miR-218-5p. Bioengineered.

[190] Han Z, Hu H, Yin M, Lin Y, Yan Y, Han P, Liu B, Jing B (2023). HOXA1 participates in VSMC-to-macrophage-like cell transformation via regulation of NF-kappaB p65 and KLF4: a potential mechanism of atherosclerosis pathogenesis. Mol Med.

[191] Si N, Meng X, Lu X, Zhao X, Li C, Yang M, Zhang Y, Wang C, Guo P, Zhang X (2020). Identification of loss-of-function HOXA2 mutations in Chinese families with dominant bilateral microtia. Gene.

[192] Chen S, Shu G, Wang G, Ye J, Xu J, Huang C, Yang S (2022). HOXA1 promotes proliferation and metastasis of bladder cancer by enhancing SMAD3 transcription. Pathol Res Pract.

[193] Xu K, Liu B, Ma Y (2019). The tumor suppressive roles of ARHGAP25 in lung cancer cells. Onco Targets Ther.

[194] Liu Z, Shen F, Wang H, Li A, Wang J, Du L, Liu B, Zhang B, Lian X, Pang B (2020). Abnormally high expression of HOXA2 as an independent factor for poor prognosis in glioma patients. Cell Cycle.

[195] Chen R, Chen B, Li D, Wang Q, Zhu Y, Li M, Wang Y, Fang S, Guo L (2019). HOTAIR contributes to chemoresistance by activating NF-kappaB signaling in small-cell lung cancer. Int J Clin Exp Pathol.

[196] Zhao Q, Zhao S, Li J, Zhang H, Qian C, Wang H, Liu J, Zhao Y (2019). TCF7L2 activated HOXA-AS2 decreased the glucocorticoid sensitivity in acute lymphoblastic leukemia through regulating HOXA3/EGFR/Ras/Raf/MEK/ERK pathway. Biomed Pharmacother.

[197] Song YP, Xian P, Luo H, Dai JY, Bai Y, Li Y, Tang XL (2022). Comprehensive landscape of HOXA2, HOXA9, and HOXA10 as potential biomarkers for predicting progression and prognosis in prostate cancer. J Immunol Res.

[198] Cui Y, Yan M, Zhang C, Xue J, Zhang Q, Ma S, Guan F, Cao W (2020). Comprehensive analysis of the HOXA gene family identifies HOXA13 as a novel oncogenic gene in kidney renal clear cell carcinoma. J Cancer Res Clin Oncol.

[199] Li J, Ye M, Zhou C (2020). Expression profile and prognostic values of HOXA family members in laryngeal squamous cell cancer. Front Oncol.

[200] Xiulin J, Wang C, Guo J, Wang C, Pan C, Nie Z (2022). Next-generation sequencing identifies HOXA6 as a novel oncogenic gene in low grade glioma. Aging.

[201] Kim KT, Lee CH, Chung CK, Kim JH (2018). Is NF2 a key player of the differentially expressed gene between spinal cord ependymoma and intracranial ependymoma?. World Neurosurg.

[202] Padam KSR, Chakrabarty S, Kabekkodu SP, Paul B, Hunter KD, Radhakrishnan R (2021). In silico analysis of HOX-associated transcription factors as potential regulators of oral cancer. Oral Surg Oral Med Oral Pathol Oral Radiol.

[203] Joll JE 2, Riley LA, Bersi MR, Nyman JS, Merryman WD. Sclerostin ablation prevents aortic valve stenosis in mice. Am J Physiol Heart Circ Physiol. 2022;323(5):H1037-1047. 10.1152/ajpheart.00355.2022 PMC9662798 36240434

[204] Mao XY, Tang SJ (2010). Effects of phenytoin on Satb2 and Hoxa2 gene expressions in mouse embryonic craniofacial tissue. Biochem Cell Biol.

[205] Cao Z, Liu C, Bai Y, Dou C, Li JM, Shi DW, Dong SW, Xiang Q (2017). Inhibitory effect of dihydroartemisinin on chondrogenic and hypertrophic differentiation of mesenchymal stem cells. Am J Transl Res.

[206] Sui Q, Chen Z, Hu Z, Huang Y, Liang J, Bi G, Bian Y, Zhao M, Zhan C, Lin Z (2022). Cisplatin resistance-related multi-omics differences and the establishment of machine learning models. J Transl Med.

[207] Suehiro Y, Suenaga S, Kunimune Y, Yada S, Hamamoto K, Tsuyama T, Amano S, Matsui H, Higaki S, Fujii I (2022). CA19-9 in combination with methylated HOXA1 and SST is useful to diagnose stage I pancreatic cancer. Oncology.

[208] Jin X, Liu X, Zhang Z, Guan Y (2020). lncRNA CCAT1 acts as a microRNA-218 sponge to increase gefitinib resistance in NSCLC by targeting HOXA1. Mol Ther Nucleic Acids.

[209] Mahajan K, Mahajan NP (2015). ACK1/TNK2 tyrosine kinase: molecular signaling and evolving role in cancers. Oncogene.

[210] Elias MH, Baba AA, Husin A, Sulong S, Hassan R, Sim GA, Abdul Wahid SF, Ankathil R (2013). HOXA4 gene promoter hypermethylation as an epigenetic mechanism mediating resistance to imatinib mesylate in chronic myeloid leukemia patients. Biomed Res Int.

[211] Barrow TM, Nakjang S, Lafta F, Bilotkach K, Woodhouse L, Junge G, Tudhope SJ, Wallis JP, Marr H, Marshall S (2021). Epigenome-wide analysis reveals functional modulators of drug sensitivity and post-treatment survival in chronic lymphocytic leukaemia. Br J Cancer.

